# Beyond Sensory Properties: Molecular Interactions of Antioxidant Flavour-Active Polyphenols Across the Food-Oral-Gut Axis

**DOI:** 10.3390/antiox15030397

**Published:** 2026-03-21

**Authors:** Inês M. Ferreira, Sara A. Martins, Leonor Gonçalves, Mónica Jesus, Elsa Brandão, Susana Soares

**Affiliations:** REQUIMTE/LAQV, Departamento de Química e Bioquímica, Faculdade de Ciências da Universidade do Porto, Rua do Campo Alegre, 689, 4169-007 Porto, Portugal; ines.ferreira@fc.up.pt (I.M.F.); sara.martins@fc.up.pt (S.A.M.); up202303110@edu.fc.up.pt (L.G.); up201405882@up.pt (M.J.); elsa.brandao@fc.up.pt (E.B.)

**Keywords:** dietary polyphenols, food matrix interactions, astringency and bitterness, extraoral TAS2Rs, bioaccessibility, oral microbiome, flavour-nutrient learning, consumer psychobiology

## Abstract

Dietary antioxidants are widely valued for their potential health benefits, but incorporating them into functional foods is not straightforward. Polyphenols are among the most abundant and important antioxidants in foods, and this review focuses on them because the same structural features linked to their health-promoting effects can also cause pronounced bitterness and astringency, ultimately limiting consumer acceptance. This review examines how these challenges are interconnected across three levels: food matrix interactions, bioavailability, and consumer psychobiology. We describe how non-covalent interactions between polyphenols, proteins, and polysaccharides can have both positive and negative effects. While these interactions may alter oral lubrication and flavour release, they also protect highly reactive bioactive compounds from gastric degradation. Furthermore, we broaden the concept of bioavailability by exploring the microbiota-mediated “colonic rescue” of polyphenols that are not released during earlier digestion. We also highlight the role of extraoral bitter taste receptors (TAS2Rs) along the gastrointestinal (GI) tract. Activation of these receptors during digestion can trigger relevant metabolic and endocrine responses, indicating that systemic absorption is not the only pathway to bioactivity. Finally, we connect these mechanisms to individual differences in food acceptance, showing that genetic factors (e.g., TAS2R38 and the salivary proteome) and psychological traits (such as neophobia and reward sensitivity) can shape rejection or flavour-nutrient learning. Overall, the successful development of functional foods will require a “sensory-by-design” approach. This strategy utilises matrix interactions strategically to improve both consumer acceptance and physiological efficacy.

## 1. Introduction

Over the past three to four decades, dietary antioxidants, including vitamins (such as vitamins C and E), carotenoids, and polyphenols, have been extensively investigated owing to their potential contribution to the prevention of chronic diseases, notably cancer and cardiovascular and neurodegenerative disorders. Within this broad group, polyphenols deserve particular attention because they are among the most abundant antioxidants in the human diet and are especially prevalent in commonly consumed plant-based foods and beverages, including tea, fruits, and derived products [1,2]. Accordingly, this review is specifically focused on polyphenols, not only because of their nutritional and biological relevance, but also because the same structural features associated with their antioxidant and health-promoting properties are frequently responsible for bitterness and astringency, thereby directly influencing consumer acceptance.

The well-known French Paradox is a classic example often cited in this context, in which a low incidence of cardiovascular diseases was associated with a regular and moderate consumption of red wine [3]. Presently, these positive effects have been related not only to the strong antioxidant capacity of polyphenols, but also to much more complex effects linked to cell signalling. From a food perspective, it is important to remember that polyphenols are a large family of plant secondary metabolites, classically divided into non-flavonoids and flavonoids (including, among others, anthocyanins and flavan-3-ols). These compounds are responsible for major sensory properties, especially colour, taste, and mouthfeel.

However, polyphenol-rich ingredients often present unpleasant sensory properties, most notably bitterness and astringency, which can strongly limit consumer acceptance [4]. This sensory “cost” is tightly connected to their chemistry: tannins, for example, a class of polyphenols, are defined by their ability to interact with and precipitate proteins, an interaction that can be at the origin of both positive and negative effects [5]. Astringency, in particular, is described as a tactile sensation involving dryness, tightening, and puckering of the oral cavity. It is a key quality parameter for red wine mouthfeel or chocolate (pleasant when balanced but perceived as harsh or “green” when excessive). On the other side, bitterness is usually an unpleasant taste, although moderate intensities are desirable in beverages like beer and coffee. Bitterness is largely mediated by bitter taste receptors (TAS2Rs), for which both receptor activation by polyphenols and strong inter-individual variability have been reported. Importantly, TAS2Rs are also expressed in extraoral tissues, especially the gastrointestinal (GI) tract, suggesting that bitter compounds may play regulatory roles in digestive and metabolic processes, reinforcing the need to look “beyond sensory properties” when designing functional foods. In this way, formulation becomes more than flavour masking. Molecular interactions in the food and in the mouth (e.g., tannin–salivary protein binding/aggregation and its modulation by carbohydrates such as pectin or gum arabic) can shape both perception and, potentially, metabolic fate, since polyphenol–protein interactions are also discussed as relevant for absorption, metabolism and bioactivity. Therefore, this review perspective is centred on the triple link between molecular interactions, bioavailability, and dietary behaviour. These three levels ultimately determine whether polyphenol fortification translates into real intake and, consequently, tangible health benefits.

This narrative review and perspective was informed by a structured search of the literature conducted in PubMed, Scopus, and Web of Science. The search was designed to identify studies most relevant to the proposed integrative framework, with particular attention to research published during the years of 2000–2026 on extraoral TAS2R signalling, microbiome-driven biotransformation, and the psychobiology of food perception and acceptance. Seminal earlier contributions were also retained where necessary to establish the mechanistic basis of polyphenol structure–activity relationships. Search terms were combined iteratively and included “dietary polyphenols”, “food matrix interactions”, “astringency”, “extraoral TAS2R”, “bioaccessibility”, “oral microbiome”, and “flavour–nutrient learning”. Retrieved articles were screened and selected through critical evaluation of their methodological robustness, scientific relevance, and direct contribution to the central themes of this review, particularly the links among molecular interactions, bioavailability, and consumer behaviour.

To provide a comprehensive overview of the literature landscape, a bibliometric analysis of the cited references was conducted using VOSviewer (v.1.6.20), visualising the interconnectedness of the topics discussed (Supplementary Figure S1). The resulting network map validates the structure of this review by revealing distinct but highly interconnected thematic clusters. The central core (light blue) highlights the chemical foundation, focused on polyphenols branching into bioavailability and metabolism (orange). From this core, two main interdisciplinary axes emerge. The oral-sensory axis (left side) links astringency, saliva, and flavour perception (yellow) with a dense network of taste, tannins, and receptor-level signalling, including bitter taste receptors, taste receptors, type 2, and calcium signalling (red). Equally, the gastrointestinal and formulation axis (right side) encompasses gut microbiota (pink), bioaccessibility and stability (dark blue), alongside digestion, nutraceuticals, and controlled release technologies (purple). Additional nodes connect these mechanisms to specific compounds like catechin and casein interactions (green), and to systemic outcomes such as oxidative stress and inflammation (top, brown). The high density of links visually reinforces the rationale of this work: molecular interactions in the food matrix and oral cavity are intrinsically linked to metabolic consequences, physiological effects, and ultimately dietary behaviour.

## 2. Molecular Interactions: The Chemistry of Polyphenol Flavour and Matrix Binding

**Figure 1:**
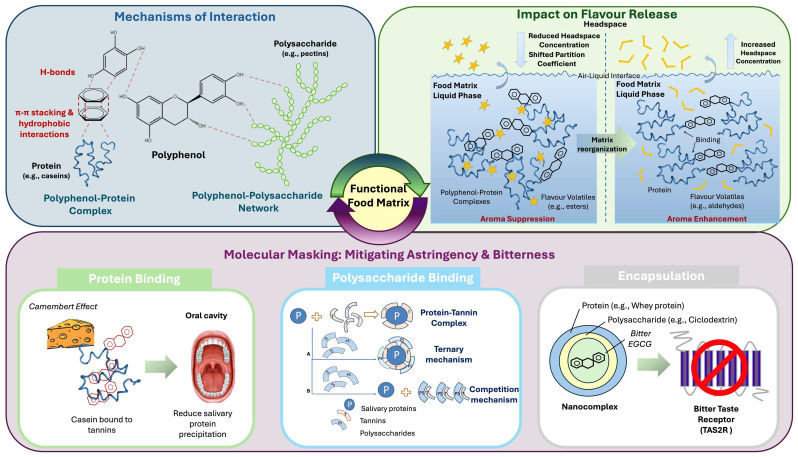
A schematic representation of molecular interactions within a functional food matrix and their subsequent impact on sensory perception. Created in BioRender. Ferreira, I. (2026) https://BioRender.com/mtrfnmm, accessed on 18 March 2026.

The presence or incorporation of bioactive compounds, such as polyphenols, into food matrices presents a fundamental challenge. The physicochemical mechanisms required to stabilise these molecules can unexpectedly alter their sensory perception. Understanding the tripartite interaction between food matrix macromolecules (e.g., proteins and polysaccharides), the bioactive partner (polyphenols), and volatile flavour compounds is critical for engineering functional foods that are simultaneously palatable, physically stable, and capable of delivering demonstrable health benefits. The upcoming sections will explore how the molecular interactions between polyphenols, proteins, and polysaccharides dictate the physical stability of food matrices. Simultaneously, they modulate flavour release, aroma volatility, and the sensory mitigation of bitterness and astringency (Figure 1).

### 2.1. Mechanisms of Interaction: Hydrogen Bonding, Hydrophobic Interactions and Supramolecular Assembly

Polyphenols are structurally predisposed to molecular associations through both covalent and non-covalent pathways. Regarding non-covalent interactions, their structural features, specifically their aromatic rings, hydroxylation patterns, and frequent galloylation, confer dual binding capacity. They can act as both hydrogen bond donors and acceptors, while simultaneously they engage in π–π stacking and hydrophobic interactions [6]. In addition to these reversible non-covalent interactions, polyphenols can also undergo irreversible covalent adduction via nucleophilic addition when exposed to oxidative conditions [7]. In general, within complex food matrices, these interactions primarily govern their association with biopolymers, such as proteins (e.g., caseins, whey proteins, plant proteins) and polysaccharides (e.g., pectin, alginate, arabinoxylans).

The immense structural diversity of these compounds dictates not only their intrinsic antioxidant capacity but also their reactivity with food matrix components. Table 1 summarises the major classes of dietary polyphenols, highlighting the structure–activity relationships (SAR) that control their redox potential and binding affinity.

#### 2.1.1. Polyphenol–Protein Interactions

When polyphenols encounter food proteins, their association relies on a specific thermodynamic and kinetic balance, largely dictated by the protein’s conformation and the ligand’s functional groups. In most cases, this is an entropy-driven process initiated by hydrophobic associations and subsequently stabilised by hydrogen bonding. Specifically, the hydrophobic benzene rings of the polyphenol scaffold drive initial interactions with aliphatic amino acid side chains (e.g., leucine), engage in CH–π interactions with the cyclic pyrrolidine ring of proline, and form π–π stacking with the aromatic rings of residues such as phenylalanine, tyrosine, or tryptophan. Following this initial contact, the phenolic hydroxyl (-OH) groups act as the primary sites for hydrogen bonding. They function as both donors and acceptors to interact with the carbonyl oxygen or amide nitrogen of the protein backbone, as well as with polar side chains. Furthermore, the presence of specific structural motifs, such as catechol groups (ortho-diphenols) or galloyl moieties (trihydroxybenzoic acid derivatives), significantly increases the density of these reactive hydroxyls and aromatic rings, substantially enhancing the overall binding affinity [7].

The interaction of polyphenols with milk caseins offers an excellent example of this mechanism. Because of their open, flexible structure and high proline content, caseins exhibit a remarkably high affinity for polyphenols. β-casein, for instance, effectively “wraps” around hydrophobic polyphenols like epigallocatechin gallate (EGCG) [19]. Molecular docking studies have identified specific binding sites, such as Pro 105, Pro 168, and Leu 180, where hydrophobic stacking stabilises the complex, while hydrogen bonds form with the peptide backbone. This intrinsic flexibility allows caseins to act as highly efficient carrier vehicles that prevent bioactive compounds from precipitating out of solution [20,21].

However, the functional consequences of these associations depend entirely on the nature of the interaction, specifically whether the bonding remains reversible or becomes covalent. Looking at caseinate–EGCG complexes, reversible non-covalent binding moderately improves the system’s antioxidant capacity and interfacial properties. Conversely, if the interaction shifts to irreversible covalent conjugation, the protein experiences a major boost in thermal stability, oxidative resistance, and emulsifying capacity. This transition from reversible association to covalent grafting reshapes protein conformation and redox distribution, demonstrating how molecular engineering of the matrix can optimise both stability and antioxidant functionality [22,23].

This relationship between structure and binding is even more pronounced in globular whey proteins (β-lactoglobulin and α-lactalbumin), which behave quite differently from the open structure of caseins. Whey proteins rely on specific and buried hydrophobic pockets to bind ligands [24]. To maximise their polyphenol-binding capacity, the protein’s tertiary structure usually needs to be partially unfolded. This is often achieved through thermal treatment, ultrasound, or pH modulation, to expose these hidden hydrophobic patches [25].

These molecular principles directly dictate the behaviour of dairy systems enriched with tea catechins or cocoa flavanols [26]. In these complex matrices, casein micelles and whey proteins form soluble or colloidal complexes that modulate both antioxidant performance and sensory traits. In vitro studies using model dairy systems show that while moderate complexation shields catechins from degradation and improves stability, excessive binding can mask radical-scavenging groups, thereby reducing measurable antioxidant activity. Crucially, processing methods like pasteurisation drive this balance. Heat-induced protein unfolding increases hydrophobic exposure and binding stoichiometry, shifting the equilibrium from reversible assemblies toward more stable, permanent aggregates [27]. These structural reorganisations directly influence flavour release, bitterness perception, and intestinal bioaccessibility, illustrating how dairy matrices can either buffer or attenuate polyphenol bioactivity depending on processing method.

Similar redox-driven complexation dynamics have been described in wine systems. In wine, the oxidation of flavanols to quinone intermediates promotes covalent bonding with nucleophilic amino acid residues. This alters colour stability and mouthfeel while simultaneously modulating antioxidant potential. These transitions exemplify how polyphenol oxidation not only restructures macromolecular assemblies but also redistributes antioxidant and pro-oxidant activities across soluble and insoluble fractions [28].

Another example of this redox-driven duality is found in honey, where polyphenols and proteins interact to form complex molecular structures that dictate its biological behaviour. Brudzynski and Maldonado-Alvarez [29] identified two distinct categories of these assemblies: “protein-type” complexes, which generally enhance antioxidant radical scavenging, and “polyphenol-type” complexes, which may instead contribute to pro-oxidant effects. These chemical transitions significantly impact honey’s medicinal qualities, often leading to a reduction in antibacterial potency and the inactivation of essential enzymes. Furthermore, environmental factors like temperature and pH further modulate these interactions, influencing the honey’s overall colour, stability, and nutritional value.

#### 2.1.2. Polyphenol–Polysaccharide Interactions

Unlike proteins, polysaccharides lack aromatic residues and therefore interact with polyphenols through mechanisms that rely more on carbohydrate chain architecture and hydration dynamics than on π–π stacking. Beyond simple physical entrapment, specific molecular forces, including hydrogen bonding, electrostatic attraction, and van der Waals interactions, contribute to the formation of stable complexes. In pectins, particularly low-methoxyl pectins, polyphenol association occurs primarily through hydroxyl–hydroxyl hydrogen-bonding networks and modulation of water structure. In these systems, the degree of esterification governs binding strength [30]. These interactions are especially relevant in plant-based matrices. Cell wall polysaccharides effectively sequester polyphenols within their structural networks, creating a diffusion-limiting microenvironment. While this might reduce immediate bioaccessibility, it physically shields the polyphenols from oxidative degradation. In this way, the polysaccharide matrix acts as a “protective cage” during harsh processing steps and later during gastric transit [31].

In fruit-based systems, specifically pectin-rich juices and purees, prolonged storage or thermal processing can trigger oxidative crosslinking, resulting in noticeable pigment darkening and structural changes in viscosity [32]. This browning reflects the transition from reversible hydrogen-bonded assemblies to irreversible, quinone-mediated covalent adducts. This pathway consumes the reactive phenolic hydroxyl groups responsible for radical scavenging. Consequently, the darkening of the matrix is typically a direct indicator of diminished antioxidant activity and a reorganisation of the product’s functional profile.

Depending on the system, polysaccharides modulate the matrix through either competitive binding or ternary complex formation. In competitive binding, they compete directly with proteins for the available polyphenols. Alternatively, during ternary complex formation, they adsorb onto and solubilise pre-existing protein–polyphenol aggregates. Rather than simply trapping polyphenols, these supramolecular assemblies dynamically reorganise the food matrix. They redistribute the bioactives across aqueous, lipid, and interfacial microphases. Ultimately, this conditions flavour partitioning, oral perception, and digestive fate [33].

### 2.2. Impact on Flavour Release: Partition Coefficients and Volatility

As polyphenols and macromolecules reorganise within the food matrix, they inevitably shift the partition coefficient (*P*) of volatile flavour compounds. This shift directly impacts the headspace concentration, and consequently, the amount of aroma reaching the olfactory receptors and sensory perception.

#### 2.2.1. Mechanisms of Aroma Suppression

Polyphenols drive this binding-induced suppression of aroma through three non-exclusive molecular mechanisms:

1. Direct hydrophobic interaction with volatile compounds
  When flavour volatiles bind to the protein–polyphenol matrix, their volatility drops, a phenomenon commonly referred to as “flavour scalping” or retention. Polyphenols, particularly higher molecular weight tannins, can directly associate with hydrophobic aroma compounds (e.g., esters and terpenes) through van der Waals forces and hydrophobic interactions. In addition, partially unfolded globular proteins such as β-lactoglobulin may generate transient hydrophobic cavities that act as reservoirs for lipophilic volatiles, further promoting retention within the matrix, as observed in in vitro binding assays [34]. While this mechanism protects labile compounds from oxidation, it simultaneously suppresses their sensory intensity, requiring higher loading to achieve the same aroma profile.
2. Indirect retention via protein–polyphenol network formation
  When polyphenols interact with proteins, especially proline-rich domains or flexible caseins, they can induce aggregation and network formation. Tannin–protein complexes increase structural heterogeneity and create supramolecular assemblies capable of entrapping aroma molecules within their interstitial spaces. This indirect retention mechanism limits volatile mobility and headspace release. For example, tannin–protein aggregation has been associated with reduced diffusion and suppressed ester volatility in model wine systems [35].
3. Viscosity-induced diffusion limitation
  Beyond molecular binding, polyphenol-induced aggregation increases matrix viscosity and alters microstructural organisation. As viscosity rises, the diffusion coefficient of aroma-active compounds decreases, slowing their migration toward the air–liquid interface [36]. This physical constraint becomes particularly relevant in protein-rich or colloidally structured systems, where polyphenol-driven crosslinking enhances bulk resistance to mass transfer.

Thermodynamically, these mechanisms reduce the chemical potential of volatile compounds in the aqueous phase. Complexation and microstructural confinement lower the air–liquid partition coefficient, decreasing headspace concentration and suppressing retronasal aroma perception. From a sensory perspective, this leads to a muted flavour, showing that polyphenol–matrix interactions can directly influence aroma perception.

#### 2.2.2. Complex Disruption and Aroma Enhancement

Interestingly, polyphenols can also help preserve certain flavours. A major cause of flavour loss in protein-rich foods is the covalent binding of aldehyde-based flavours (like vanilla or fruit aromatics) to the ε-amino groups of lysine residues via Schiff base formation [37]. When polyphenols are introduced, they compete for these exact binding sites or sterically block the reactive amino groups. By preventing this covalent trapping, polyphenols ensure that a higher concentration of free aldehyde remains available to volatilise into the headspace, effectively rescuing specific aroma notes from being lost to protein conjugation [37,38].

### 2.3. Molecular Masking: Intrinsic Strategies to Mitigate Bitterness and Astringency

Astringency arises primarily from the interaction of polyphenols with salivary proteins and other oral components, leading to protein precipitation and loss of lubrication. This perception can be reduced through target binding strategies, where polyphenols are pre-complexed with food proteins such as caseins or gelatine during processing. By occupying reactive phenolic sites, these proteins limit the ability of polyphenols to bind oral constituents. This phenomenon is exemplified by the “Camembert effect”, where cheese proteins preferentially bind wine tannins, decreasing perceived astringency [39]. Additional masking mechanisms include microencapsulation within protein–polysaccharide complexes, which act as a diffusion-limiting barrier that operates at both molecular and microstructural scales. In the case of whey protein/β-cyclodextrin-EGCG nanocomplexes [40], whey protein isolate provides a flexible binding scaffold through hydrophobic interactions and hydrogen bonding. Meanwhile, β-cyclodextrin contributes a hydrophobic cavity capable of forming inclusion complexes with EGCG. This dual encapsulation strategy reduces the free fraction of EGCG in the aqueous phase, thereby limiting its immediate interaction with salivary proteins and TAS2R bitter receptors. Polysaccharides such as pectins and arabinoxylans provide another mechanism by interfering with polyphenol–salivary protein aggregation [41]. Rather than preventing binding entirely, they shift interactions towards soluble complexes that reduce friction and perceived astringency. For bitterness, triggered by TAS2R receptors activation, hydrocolloids (like xanthan gum) offer a steric masking route. By encapsulating bitter compounds and increasing the bolus viscosity, these polysaccharides severely restrict the diffusion of bitter molecules to the taste buds [42]. Additionally, lipids contribute through phase partitioning; because many polyphenols are hydrophobic, they preferentially localise within fat droplets, lowering their availability to interact in the oral cavity, reducing astringency and bitterness perception [43].

## 3. Molecular Interactions: The Biochemistry of Polyphenol Flavour Inside the Oral Cavity

The oral cavity is a highly complex and dynamic bioreactor in which anatomical heterogeneity, fluid mechanics, and biochemistry converge over very short timescales. During eating, foods are exposed to spatially distinct epithelial surfaces both gustatory and non-gustatory. They encounter continuously renewed salivary and mucosal films, rapidly changing dilution and shear conditions, and diverse, site-structured microbiota. Together, these factors generate a heterogeneous microenvironment where local pH, ionic strength, enzymatic activity, and interfacial adsorption can vary across regions and through time. This variability conditions both flavour perception and the physicochemical form in which bioactive and polyphenols are ultimately swallowed [38].

Whole saliva is a dynamic biological fluid that initiates the oral phase of digestion and constitutes the first biochemical environment encountered by polyphenol-rich foods. It is a composite of secretions from the major and minor salivary glands, mixed with gingival crevicular fluid and a variable microbial and cellular component. Its physicochemical properties (flow rate, ionic strength, viscosity, pH, buffering capacity) and composition shift rapidly with stimulation. This means that the “oral medium” is not constant but adapts to the act of eating [44]. At the molecular level, saliva is predominantly water, yet its functional impact is driven by electrolytes (notably bicarbonate/phosphate buffering systems, calcium, and other ions), low-molecular-weight metabolites, and a dense proteome comprising mucins, proline-rich proteins (PRPs), statherin, histatins, cystatins, amylase, immunoglobulins, and multiple defence-related enzymes [45,46]. Functionally, these constituents provide lubrication, enamel protection, and antimicrobial activity. Crucially for food perception, they also ensure the continuous renewal of interfacial films (both mucosal and enamel pellicle) coating oral tissues [47,48].

**Figure 2:**
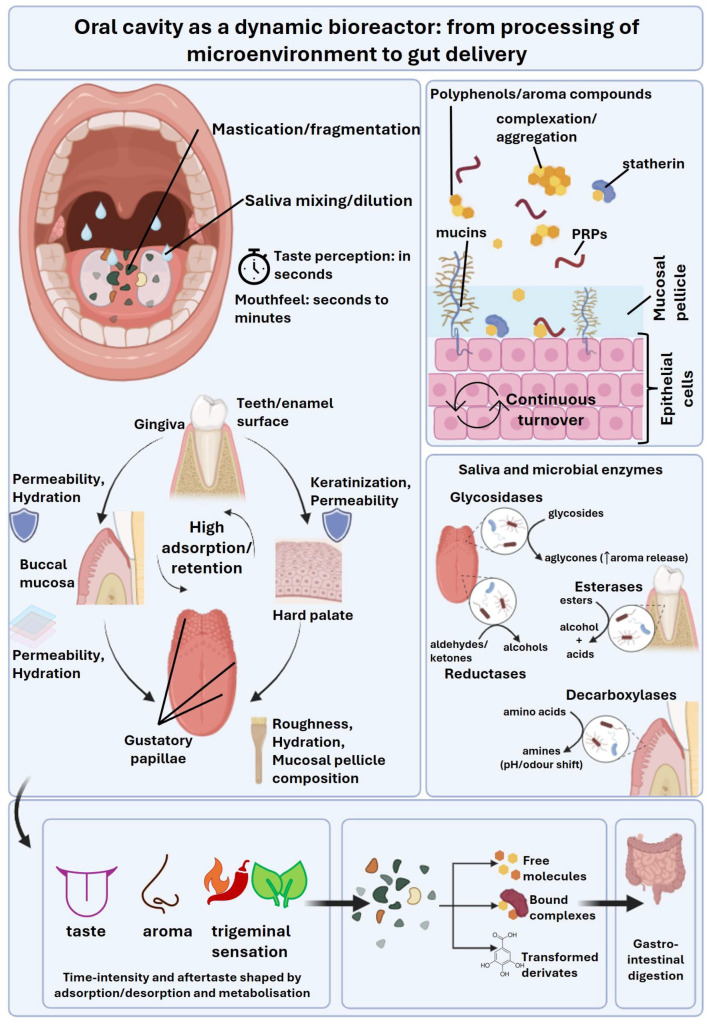
The oral cavity as a dynamic bioreactor. Mastication rapidly fragments food and mixes it with saliva, initiating taste and mouthfeel perception. The oral environment is heterogeneous. Different surfaces exhibit distinct permeability and hydration, which influence compound adsorption and retention. Polyphenols and aroma compounds continuously interact with the mucosal pellicle and salivary proteins. Simultaneously, host and microbial enzymes biotransform substrates, altering local pH and aroma release. Together, these mechanical, physicochemical, and enzymatic processes shape the time–intensity profile of oral sensations. Ultimately, they determine whether bioactives reach the GI tract as free compounds, bound complexes, or transformed derivatives. Symbol: ↑, increased. Created in BioRender. Ferreira, I. (2026) https://BioRender.com/3albew3, accessed on 18 March 2026.

Flavour emerges from the integration of three sensory channels alongside tactile mouthfeel. These channels include taste (receptor-mediated detection of non-volatile solutes), aroma (ortonasal perception through direct smelling and retronasal perception of volatiles released during mastication), and trigeminal or chemesthetic sensations (irritation, cooling, heat, tingling). Saliva modulates all three through perireceptor processes. It solubilises, metabolises, and transports tastants to taste pores. It also shapes diffusion and clearance kinetics, modulates volatile partitioning and transport in the oral headspace, and establishes the lubrication microenvironment that governs frictional and tactile sensations [38]. For functional foods enriched with polyphenols and other bioactives, these salivary processes become a mechanistic bridge. They link sensory acceptance (and thus dietary choice) and the physicochemical form in which compounds are swallowed. This ultimately has downstream consequences for bioaccessibility, bioavailability and health outcomes (Figure 2).

### 3.1. Saliva-Driven Binding and Mouthfeel: Protein Interactions, Lubrication Changes, and Astringency/Bitterness Modulation

Astringency is best framed as a multifactorial oral tactile phenomenon in which biochemical interactions translate into altered interfacial mechanics [4,49]. Polyphenols, particularly tannins, exhibit a strong propensity to associate with salivary proteins. These classically include PRPs, alongside other salivary proteins and peptides. This association forms soluble complexes and, under many conditions, larger aggregates. These events can reduce the concentration of lubricating proteins and perturb the structure of the salivary and mucosal pellicle, increasing friction between oral surfaces during movement. In parallel, polyphenols may interact directly with mucosal components, contributing to mucoadhesion and local pellicle remodelling. The perceived outcome (dryness, roughness, puckering) depends on the balance between complexation/aggregation kinetics, the individual salivary proteome, and the mechanical context of oral movements (tongue–palate and cheek–tooth contact). This helps rationalise why astringency intensity and “quality” vary markedly between individuals and between matrices even at similar polyphenol levels.

Similarly to polyphenol–protein interactions, polyphenol–salivary proteins also exhibit marked structure–activity relationships [4,49,50]. In general, increasing molecular weight and degree of polymerisation (e.g., proanthocyanidin oligomers/polymers) enhances multivalent binding to PRPs and mucins. This occurs via cooperative hydrogen bonding, aromatic (π–π) stacking, and hydrophobic interactions, favouring precipitation and thereby intensifying lubrication loss and perceived astringency. Notably, many of these same structural determinants also govern intrinsic antioxidant capacity. Higher phenolic hydroxyl density, catechol (ortho-dihydroxyl) or galloyl/pyrogallol motifs, and extended conjugation/planarity promote electron or hydrogen-atom donation. Furthermore, they also stabilise phenoxyl radicals by resonance and increase transition-metal chelation. Consequently, features such as galloylation and B-ring catechol substitution frequently increase both salivary protein affinity and redox/chelating activity. Conversely, glycosylation and O-methylation typically attenuate both by reducing accessible hydroxyls, disrupting planarity, and diminishing aromatic stacking. This coupling implies a mechanistic trade-off relevant to functional food design. Polyphenols optimised for high in vitro antioxidant reactivity are often those most prone to strong oral protein binding. This results in downstream consequences for sensory outcomes and for biological performance through altered solubility, colloidal state, and oral-to-gastrointestinal release kinetics.

Bitterness, by contrast, is mediated by TAS2Rs, which are G protein-coupled receptors (GPCRs). Humans express 26 different TAS2Rs, which are activated when agonists reach TAS2R on taste cells through a diffusion-limited and saliva-conditioned pathway. Saliva can attenuate or reshape bitterness by (i) binding bitter ligands and lowering free concentration near the taste pore; (ii) altering speciation/ionisation states that influence receptor affinity; and (iii) changing residence time via clearance kinetics. Importantly, astringency and bitterness frequently co-occur in polyphenol-rich foods and are not independent. Salivary binding and pellicle changes can shift both tactile mouthfeel and the time–intensity profile of bitter receptor stimulation. From a formulation standpoint, this implies that “bitterness control” is not only a receptor problem but also an interfacial chemistry and mass-transfer problem.

### 3.2. Enzymes and pH Reshape Taste and Aroma: Oral Biotransformation by Host and Microbiota

The oral cavity is also an enzymatically active reactor operating under buffered conditions. Salivary buffering (mainly bicarbonate/phosphate systems) stabilises pH within a physiologically relevant range during eating. This regulation directly influences polyphenol ionisation, metal chelation states, and overall reactivity. Consequently, these physicochemical shifts can alter both binding equilibria (such as protein complexation) and receptor activation. Equally important is the oral microbiota, which supplies additional enzymatic capacity with high relevance to aroma formation.

Salivary enzymes can be grouped by functional classes, with human and microbial sharing similar classes, as summarised in (Table 2).

Microbial communities are individualised, site-dependent (see next section), and markedly impacted by food choices. Consequently, the capacity for oral biotransformation can vary between individuals, contributing to perceptual diversity and potentially to differences in the chemical form of bioactives entering the upper GI tract [62].

Critically, the food matrix regulates oral enzyme action. Substrate accessibility is shaped by microstructure (emulsions, gels, protein networks), viscosity, and competitive binding. For instance, polyphenol–protein complexation can shield phenolics from enzymatic transformation or redirect them into alternative reaction pathways. Similarly, fats can sequester hydrophobic aroma compounds and some bitter ligands, while polyphenol–polysaccharides interaction can alter diffusion and residence time. Thus, oral enzymatic effects on flavour are not solely enzyme-dependent; rather, they are highly matrix-conditioned.

### 3.3. Oral Microstructure Governs Release Kinetics and Local Exposure: Heterogeneous Surfaces, Adsorption, and Delivery to the Gut

Oral tissues are heterogeneous, which is crucial for both flavour sensation and bioactive fate. Gustatory epithelia (taste buds within specialised papillae) coexist with extensive non-gustatory epithelia (buccal, labial, gingival, hard palate regions). These distinct zones differ significantly in keratinisation, permeability, surface roughness, hydration, and pellicle composition. These differences create region-specific interfacial environments that can promote differential adsorption/retention and/or metabolization of dietary compounds [63]. In practical terms, polyphenols and aroma compounds may partition differently onto mucosal pellicles with distinct protein–lipid–mucosal pellicle compositions. Therefore, the same molecule can exhibit different residence times depending on whether it contacts, for example, the tongue dorsum versus the buccal mucosa [64].

This spatial heterogeneity intersects with site-specific microbiota. The tongue, cheeks, and dental surfaces harbour distinct microbial communities with different metabolic potentials. Therefore, both adsorption phenomena and microbial biotransformation can be location-dependent, adding a spatial dimension to flavour evolution and to the chemical “pre-processing” of bioactives.

Crucially, these region-specific oral interfaces are not static; they change throughout the lifespan. Their temporal dynamics further modulate adsorption, release kinetics, and downstream delivery to the gut. The mucosal surface is continuously coated by a mucosal pellicle. This protein- and mucin-rich film forms rapidly from saliva and concentrates protective constituents (e.g., mucins, statherin, immunoglobulins). This process creates a dynamic “reaction/retention layer” that can reversibly bind polyphenols and aroma compounds and prolong local exposure. In parallel, the underlying epithelium undergoes rapid turnover and desquamation, meaning that surface composition (glycoproteins, lipids, barrier proteins) and permeability can shift over days. These shifts alter both the availability of binding sites and the extent of compound uptake or wash-off [65]. Longer-term remodelling, driven by inflammation, smoking/irritants, ageing, or shifts in diet, can further reshape epithelial–stromal architecture and barrier function. This has been highlighted by single-cell atlases of human oral mucosa across various disease states. Such remodelling has direct consequences for how polyphenols partition into films, persist on surfaces, and are subsequently swallowed and presented to the GI tract [66]. Nutrition also plays a vital role, as vitamin deficiencies and high-sugar diets impair tissue regeneration and decrease salivary flow, leading to enamel hypomineralisation and infections.

Within this structured system, mastication dynamics govern kinetics. Fragmentation increases surface area and disrupts food matrices. Simultaneously, saliva incorporation dilutes solutes and sets binding equilibria, while oral warming increases diffusion and volatility. Together, these variables determine three key factors: (i) how rapidly polyphenols are liberated from the food matrix; (ii) how long they remain in contact with oral proteins, mucosal pellicles, and epithelia; and (iii) the extent to which they are swallowed as free molecules versus bound complexes or transformed derivatives. This is where oral processing becomes directly relevant to bioaccessibility: the oral phase can alter the distribution of molecular forms delivered to the GI tract, influencing subsequent release, stability, and absorption. At the same time, these kinetics shape time–intensity sensory profiles that directly feed back into liking, intake, and long-term adherence to functional foods.

### 3.4. Persistence and Aftertaste: Adsorption Reservoirs and Slow Desorption from Interfacial Films

Many sensations associated with polyphenol-rich foods are temporally extended. Persistence arises when flavour-active molecules adsorb to oral surfaces or partition into salivary/mucosal films, forming a reservoir that desorbs slowly after swallowing. For polyphenols, prolonged dryness or roughness can reflect continued pellicle disruption, slow rearrangement of protein–polyphenol complexes, and sustained changes in lubrication. Regarding bitterness and aroma, lingering effects can result from slow release of ligands/volatiles from mucosal pellicles or from protein/lipid-associated phases within the mouth. These aftereffects are not merely sensory nuisances. Instead, they can be decisive drivers of acceptance and repeated choice, thereby shaping dietary exposure. Simultaneously, prolonged oral retention increases local contact time, potentially enhancing local interactions (protein binding, mucosal association) while modulating the fraction and form of compounds that proceed to the gut.

Across all four mechanisms, the central point remains the same: the oral cavity is a chemically and mechanically active environment. It conditions both what people choose to consume and specific form of polyphenol dose delivered for absorption. Ultimately, this links molecular interactions in the mouth to bioaccessibility and, ultimately, to health outcomes.

## 4. Impact on Bioaccessibility and Bioavailability

The journey of dietary polyphenols through the GI tract is governed by a complex interplay of physiological barriers and molecular interactions. Building upon the structural and oral dynamics previously discussed, this section explores how these matrices’ associations extend their influence far beyond sensory perception. Here, we examine how these structures fundamentally dictate the bioaccessibility, systemic bioavailability, and novel luminal signalling of bioactive compounds as they transit from the gastric environment to the lower gut.

### 4.1. Protective Role of the Food Matrix

The gastric phase represents a critical physicochemical bottleneck in the transit of dietary polyphenols. The highly acidic environment (pH 1.5–3.0), the presence of proteolytic enzymes (pepsin), and high ionic strength collectively drive the chemical degradation of labile bioactive compounds before they reach the main absorptive epithelium of the small intestine [67,68,69]. While molecular interactions in the oral cavity are often discussed in the context of sensory perception and suppression (e.g., masking bitterness), recent high-impact investigations using simulated in vitro digestion models reveal that these same interactions—specifically the sequestration of polyphenols within protein [69,70], polysaccharide [41,71,72], or lipid compounds [73]—confer a vital protective advantage during gastric digestion. This shielding effect is not merely a passive retardation of release but a result of active molecular stabilisation mechanisms [69,74], including steric hindrance [70], redox buffering [69], and interfacial engineering [73]

#### 4.1.1. Protein-Based Entrapment and Structural Modification

Proteins serve as the primary dietary vehicles for polyphenols [75,76]. However, their efficacy as gastric protectants is strictly governed by the interplay between binding affinity and proteolytic stability [69,73]. Native globular proteins alone often provide insufficient protection because they are rapidly hydrolysed by pepsin, exposing bound antioxidants to the acidic bulk phase [69,70,77]. Recent mechanistic insights suggest that specific structural modifications can exploit protein unfolding to enhance hydrophobic sequestration, effectively converting “flavour masking” into “acid masking” [77]. Fundamental work using single-molecule force microscopy has demonstrated that polyphenols such as EGCG induce a structural collapse in proteins like β-casein [78]. This binding significantly reduces the protein’s radius of gyration, indicating a “hydrophobic wrapping” effect where the protein polymer curls around the polyphenols. This compaction entropically stabilises the complex and physically excludes the acidic solvent from the polyphenol’s reactive hydroxyl groups, retarding auto-oxidation. Wang et al. [69] further advanced this concept in an in vitro model by coupling ultrasound-induced unfolding with steric shielding to encapsulate (-)-gallocatechin gallate (GCG). By exposing buried hydrophobic regions of ovalbumin (OVA) via ultrasound and subsequently glycating the protein with pectin, they engineered a “layer-by-layer” shield. Under simulated gastric conditions, this glycated complex (G-UOVA) functions synergistically: the hydrophobic burial tightly secures the polyphenol core, while the external polysaccharide chains provide steric hindrance against pepsin access, preserving the bioactive form until intestinal transit. Thus, the same strong hydrophobic affinity that suppresses bitterness perception in the oral cavity emerges as the fundamental physicochemical prerequisite for chemical survival through the gastric bottleneck [69].

On the other side of this strategy, the same compaction and surface shielding that protect polyphenols can lower the protein’s bioaccessibility. It achieves this by slowing gastric-intestinal proteolysis and decreasing the rate (or extent) of amino acid and bioactive peptide release. In extreme cases, advanced glycation-type modifications combined with polyphenol association may also alter the profile of digestion products. This will be further discussed in Section 4.2.2.

Additionally, a complementary physiological example illustrates that protein-based entrapment can also begin in the oral cavity. Here, saliva act as an initial “binding/precipitation gate” for complex tannins, as discussed in Section 3. In an in vitro wine-ingestion model, Soares et al. [79] showed that red-wine procyanidin fractions form (in)soluble complexes with salivary protein. However, their gastric fate is strongly dependent on the degree of polymerisation. Complexes involving low-mean degree of polymerisation (mDP) species (monomers to trimers) were largely disrupted under simulated gastric conditions (pepsin, pH ~1.7), releasing procyanidins into the supernatant. In contrast, higher oligomers (tetramers to pentamers) generated more resistant, largely insoluble aggregates that persisted through digestion. Notably, the most polymerised fractions were also the most effective at depleting key salivary targets, particularly statherin and acidic PRPs (aPRPs). Statherin-associated complexes appeared especially stable, implying that oral protein binding can selectively reduce the bioaccessible fraction of larger procyanidins reaching the GI tract. This mechanism must be considered alongside engineered “acid-masking” protein carriers when predicting net polyphenol delivery.

Salivary sequestration of highly polymerised procyanidins may be viewed as a protective pre-gastric filter. The precipitation of tetramer/pentamer-rich fractions into insoluble protein–tannin networks can lower the load of highly reactive tannins entering the upper GI tract, potentially mitigating digestive-enzyme inhibition. Importantly, however, the nature and stability of these complexes are likely individual-dependent. This is attributed to the relative abundance and isoform distribution of key salivary binders (notably statherin versus aPRPs and glycosylated PRPs (gPRPs)) varying across individuals. Given that statherin–procyanidin complexes appear particularly stable and less readily dissociated than PRP-associated complexes, individuals with statherin-rich profiles may retain/precipitate a larger fraction of high-mDP tannins through gastric transit. Conversely, PRP-dominant profiles may favour complexes that release more readily under stomach conditions. Consequently, the fraction ultimately liberated (small intestine) versus diverted to the colon (for microbiota-driven catabolism into smaller phenolics) may differ substantially between individuals, even with identical tannin exposure.

#### 4.1.2. Polysaccharide Reinforcement: From Interfacial Engineering to Bulk Matrices

While hydrophobic sequestration within protein cores provides a molecular-level shield [75], the stability of lipophilic antioxidants dispersed in emulsions is governed by the supramolecular architecture of the oil–water interface [80]. Native interfacial proteins are susceptible to pepsin protease activity. This typically triggers flocculation and coalescence, releasing the bioactive payload into the acidic gastric fluid. To counteract this, interfacial engineering utilises polysaccharide coatings as secondary barriers that reinforce the primary protein layer against gastric stressors.

The primary mechanism driving this protection is steric hindrance. In the case of curcumin stabilisation, Aguilera-Garrido et al. [73] showed that native bovine serum albumin emulsions retained only 25% of the bioactive compound. In contrast, the electrostatic deposition of a hyaluronic acid (HA) shell boosted retention to 85% by restricting pepsin’s access to the interface.

Furthermore, certain interfaces exhibit pH-responsive “smart” behaviour. Duan et al. [70] engineered ternary complexes utilising whey protein isolate, seaweed polyphenols, and sodium alginate. Here, gastric acidity triggers the protonation of alginate carboxyl groups, transitioning the polymer from a soluble coil to an insoluble, gel-like shell. This acid-induced tightening locks the polyphenol–protein complex within a rigid network, reducing the diffusion of hydronium ions and oxidants into the droplet core.

This contradicts the traditional view of gastric processing as a purely degradative phase. Instead, it frames the stomach as a pH-precision functional stage. In this environment, acid-driven interfacial and matrix interactions promote in situ self-assembly into more cohesive, diffusion-limiting protective architectures.

Beyond functioning merely as electrostatically deposited secondary layers at the oil–water interface, polysaccharides can also be engineered as the primary structural-encapsulating matrix. This fundamentally shifts the protective mechanism from localised interfacial shielding to bulk volumetric entrapment [81,82]. In systems such as hydrogel beads, microgels, and complex coacervates, the lipophilic payload is embedded within a dense biopolymer network. This network is typically formulated via ionic gelation (e.g., calcium-crosslinked alginate or pectin) or protein–polysaccharide electrostatic complexation [76,83,84]. These macroscopic and mesoscopic matrices exhibit profound pH-responsive behaviour. In the gastric environment, the tightly crosslinked, sterically hindered polymer networks severely restrict the internal diffusion of pepsin and gastric fluids, providing exceptional resistance to both enzymatic degradation and antro-pyloric shear stress [85,86,87].

Upon transitioning to the neutral pH and distinct ionic composition of the small intestine, these matrices undergo controlled structural disassembly, driven by electrostatic repulsion and ion exchange processes [88]. This programmed uncoiling facilitates a diffusion-controlled release of intact lipid droplets directly into the duodenal lumen. Consequently, optimised microgel systems frequently demonstrate a multifold increase in quantitative bioaccessibility compared to unprotected emulsions [89]. Ultimately, while these polysaccharide architectures dictate gastric survivability, the intrinsic physicochemical properties of the encapsulated lipid phase—such as triacylglycerol chain length (medium-chain versus long-chain triglycerides, MCT vs. LCT), degree of unsaturation, and lipid crystallinity—fundamentally govern subsequent intestinal lipolysis kinetics and the solubilisation capacity of the mixed micellar phase [90,91,92]. Therefore, coupling a robust, pH-responsive polysaccharide matrix with a highly solubilising liquid LCT core is critical. This combination is essential for maximising the ultimate micellarisation efficiency (due to the larger hydrophobic core of LCT-derived micelles) and the final bioaccessibility of highly lipophilic antioxidants.

#### 4.1.3. The “Natural Matrix” Effect: Whole Food vs. Purified Extracts

Engineered encapsulation systems essentially mimic the natural protective structures found within whole foods, commonly referred to as the food matrix. The complex, heterogeneous architecture of this matrix functions as a formidable physicochemical barrier against gastric degradation [80,93]. The current literature challenges the reductionist approach of utilising purified extracts, suggesting that the removal of dietary fibre, endogenous proteins, and hydrocolloids strips polyphenols of their primary defence system [71,94].

Kumkum et al. [71] established a protective hierarchy in acai berry formulations, where the whole fruit significantly outperformed purified extracts in preventing anthocyanin degradation. Specifically, purified extracts suffered higher degradation (yielding only 77% recovery) compared to the whole fruit (94% recovery). The mechanistic driver appears to be the natural network imposed by cellulose and pectin molecules. This network limits the diffusion of gastric oxidants and enzymes into the particulate matter [71]. Similarly, Yvonne et al. [72] showed that during simulated in vitro GI digestion, the stability of labile betaxanthins (e.g., indicaxanthin) in red prickly pear juice is strictly dependent on the viscosity of the continuous phase. The presence of natural mucilage (arabinogalactan/pectin) increases digesta viscosity, thereby reducing the proton diffusion coefficient and providing metal-chelating capacity—a dual protection that is lost in clarified juices. Furthermore, this protection persists through thermal processing and within solid food matrices. Bavaro et al. [95] highlighted that in artichoke-enriched pasta, the gluten protein network and gelatinised starch effectively encapsulate phenolic acids. Although cooking initially reduces the content of free polyphenols, the gastric phase reveals a “rebound” in bioaccessibility. This phenomenon is attributed to the acid-hydrolytic release of compounds that were previously protected within the starch–gluten matrix, effectively functioning as a heat-stable delivery system. Ultimately, this structural resilience imposes a critical pharmacokinetic trade-off. The steric barriers required to promote gastric protection inevitably hinder the initial interfacial access of duodenal lipases and bile salts [73,95]. Consequently, both the “foodome” and engineered matrices function effectively as delayed-release systems. This reframes bioaccessibility not as a static chemical constant, but as a dynamic variable governed by matrix release kinetics.

### 4.2. Release Kinetics and Intestinal Absorption: The Solubility–Permeability Interplay

The transition of a bioactive and antioxidant compound from a “flavour/bioactive protected-complex” in the oral cavity to a bioaccessible molecule in the small intestine represents a critical shift in physicochemical requirements. While the oral and gastric phases prioritise molecular sequestration—to mask sensory attributes [96] and prevent acid-catalysed degradation [97]—the intestinal phase demands efficient release. The central dogma for this stage is the solubility-permeability interplay. Effective transepithelial transport requires a compound to be soluble in the aqueous lumen, yet sufficiently lipophilic to traverse the apical membrane [98]. Consequently, the impact of the food matrix on bioavailability is defined by a correlation between maintaining solubility and ensuring a sufficient free fraction of the molecule for diffusion.

#### 4.2.1. From Gastric Processing to Intestinal Release

The release kinetics of flavour-active compounds is fundamentally initiated by the disintegration of food microstructures within the gastric compartment (upon physical and chemical digestion processes). As established by Kong and Singh [99], the specific disintegration mechanism dictates the interfacial surface area available for downstream enzymatic hydrolysis. In proteinaceous matrices, particularly hydrogels and high-protein digesta, gastric acidity and ionic strength drive osmotic swelling, a physicochemical state that governs the rate of matrix erosion [100,101]. As polyphenols transit from the stomach to the small intestine, their trajectory is dictated by whether the protective macromolecular assemblies remain stable or are successfully dissociated. This dynamic transition is strictly modulated by three key factors: local pH shifts, enzymatic activity, and the intrinsic composition of the food matrix. While the gastric phase relies on matrix resilience against acid and pepsin to prevent premature degradation, the intestinal phase demands a functional reversal. Here, the transition to a neutral pH, combined with the influx of bile salts and pancreatic enzymes, serves as the primary environmental trigger for dissociation. However, this requirement for release introduces a kinetic paradox: encapsulation strategies designed for gastric protection can inadvertently hinder intestinal bioaccessibility Aguilera-Garrido et al. [73] demonstrated this trade-off in curcumin-loaded lipid nanocapsules. While a HA shell successfully mitigated gastric degradation, it significantly delayed the subsequent release of free fatty acids and curcumin during the initial intestinal phase. While essential for gastric stability, such steric exclusion of lipases and bile salts can shift the release profile distally, potentially bypassing the primary absorptive windows of the proximal small intestine.

#### 4.2.2. Solubility–Permeability Interplay and Transepithelial Transport

If a “flavour/bioactive protected-complex” fails to dissociate in the small intestine, the penalty is often greatest for proteins. Strong polyphenol–protein affinity (e.g., tannins/catechins binding PRPs), polyphenol-mediated compaction/aggregation, or added shielding layers (such as pectin or alginate coatings) can mask cleavage sites. Consequently, this slows pepsin and trypsin access, decreasing the rate and extent of proteolysis. In practical terms, complexes such as tea catechins-casein/β-lactoglobulin [102,103] or condensed tannin-sorghum/legume proteins [104] can behave as digestion-resistant aggregates. This shifts the digestive output from absorbable amino acids and small peptides towards larger fragments. Consequently, this increases the fraction of protein that reaches the colon. This outcome is undesirable when the protein is intended as a high-quality source of amino acids. Furthermore, it can lower effective protein quality by reducing amino acid bioaccessibility. It also alters the bioactivity profile of released peptides (e.g., generating fewer antihypertensive or antioxidant peptides during digestion) and modifies physiological responses such as satiety signalling and allergen epitope exposure. Crucially, this digestion-resistant entrapment equally impairs the bioactive potential. While protein complexation predominantly protects polyphenols against gastric chemical degradation, it concurrently limits their intestinal release. As evidenced by the previously described studies [104,105], when these supramolecular aggregates resist pancreatic proteolysis, the polyphenols remain sequestered within the matrix debris. Consequently, the free fraction of polyphenols available for transepithelial absorption is severely reduced, directly compromising their bioaccessibility and systemic bioavailability.

By contrast, incomplete release is often less problematic for polysaccharides, because many are intrinsically non-digested in the upper GI tract. If polyphenol remains associated with pectin, alginate, inulin, β-glucan, or resistant starch, the complex can still be physiologically useful. It functions as a fermentable substrate for the colonic microbiota, as detailed in Section 4.3.

Upon entry into the duodenum, bile salts function as biological surfactants, forming mixed micelles essential for solubilising lipophilic bioactives. While this interaction increases apparent solubility, it introduces a critical pharmacokinetic trade-off known as the “permeability penalty.” As described by Beig et al. [105], the intestinal membrane is impermeable to large micellar complexes. Only the free fraction of the compound drives passive diffusion. Consequently, excessive binding affinity creates a kinetic trap. Even with a high total concentration in the lumen, the bioactive remains sequestered within micelles or matrix debris, leaving a negligible free fraction available to drive epithelial transport [98,105]. This phenomenon represents the “free fraction limit,” where protective complexes effectively render the payload biologically inert regarding passive diffusion unless they dissociate rapidly at the epithelial surface [97]. The functional role of these mixed micelles, whether they act as facilitators or sequestrants, depends strictly on the reversibility of the polyphenol–micelle complex. Under conditions of reversible complexation, micelles facilitate the absorption of highly lipophilic compounds by acting as shuttles that transport their hydrophobic content across the aqueous unstirred water layer to the brush border [106]. Conversely, if the binding affinity to the micellar core is excessively strong or irreversible, micelles act as sequestrants that trap the bioactive compound, thereby limiting the availability of the free fraction required for epithelial transport [100,106]. Beyond these physicochemical dynamics, net bioavailability is commonly interrogated using advanced transepithelial co-culture systems. For example, Transwell Caco-2 intestinal barriers are often complemented with mucus-secreting HT29-MTX cells and coupled to immune-responsive THP-1-derived macrophages. These models capture how epithelial transport, barrier function, and inflammatory signalling collectively modulate the fraction of bioactives that becomes biologically available [107].

High bioaccessibility does not strictly correlate with high bioavailability, as transport is often restricted by xenobiotic extrusion mechanisms. Recent work by Guo et al. [106] using Caco-2 cell monolayers illustrates that the uptake of catechins and chlorogenic acid is governed by the ability of the polyphenol mixture to competitively inhibit apical efflux pumps (P-glycoprotein (P-gp) and Multidrug Resistance-Associated Protein 2 (MRP2)), effectively overcoming the cell’s natural rejection barrier. Furthermore, understanding the relationship between intestinal bioavailability and systemic bioactivity requires considering post-absorptive metabolic processes. Once the free fraction of polyphenols successfully traverses the intestinal epithelium, these compounds undergo extensive Phase II metabolism, predominantly glucuronidation, sulfation, and methylation, within the enterocytes and subsequently in the liver [108,109,110]. Consequently, the circulating metabolites often exhibit significantly altered redox potentials, half-lives, and biological activities compared to their native aglycone forms present in the food matrix [111]. This extensive first-pass metabolism highlights that the successful intestinal absorption of a parent compound does not necessarily equate to high systemic bioactivity of that same native structure. This pharmacokinetic reality reinforces the physiological importance of alternative pathways, specifically luminal signalling via extraoral TAS2Rs [112] and microbiota-driven colonic biotransformation, which are independent of classical systemic absorption [113].

In summary, the optimal “flavour/bioactive protected-complex” is fundamentally release-responsive. It leverages strong molecular interactions (e.g., with PRPs or polysaccharides) for both sensory masking and gastric protection. However, it critically relies on environmental triggers—such as pH shifts, bile salt displacement, or enzymatic hydrolysis—to dissociate in the small intestine. The failure of a complex to release its payload transforms a potential systemic bioactive into a non-absorbable colonic substrate. Therefore, designing functional foods requires a precise physicochemical balance: exploiting the solubility advantage of encapsulation while avoiding the permeability penalty of excessive binding.

### 4.3. Microbiota Interaction: Colonic Biotransformation as a Determinant of Late-Stage Bioactivities

The free fraction limit and matrix entrapment described in Section 4.2 effectively restrict small-intestinal absorption to a minor fraction (often 5–10%) of total polyphenol intake. Consequently, the gut microbiota functions not merely as an ecological niche, but as a critical, late-stage pharmacocompetent organ. It facilitates the secondary release and molecular simplification of the non-absorbed fraction, particularly for non-extractable polyphenols (NEPPs) intimately bound to dietary fibre [114,115].

#### 4.3.1. Mechanistic Logic of Colonic Liberation

The transition of polyphenols from the small intestine to the colon marks a critical shift from host-driven digestion to microbial-mediated biotransformation. High-molecular-weight tannins, alongside polysaccharide-entrapped and protein-bound polyphenols, arrive in the large intestine relatively unmodified due to their structural complexity. In this environment, the microbiota exerts a synergistic effect termed “release-responsive” action. Resident microbes first ferment the insoluble dietary fibre and polysaccharide network, effectively dismantling the physical barriers that precluded earlier absorption [114,116]. Fermentation can increase short-chain fatty acid production and selectively enrich taxa that utilise these glycans (a prebiotic-like effect). Simultaneously, microbial enzymes can progressively liberate and transform the bound polyphenols into smaller phenolic acids that may be absorbed later or act locally in the colon.

This rigorous enzymatic sequence is designed to reduce molecular weight and improve membrane permeability. The initial rate-limiting step is often deglycosylation. While the host possesses limited hydrolytic capacity via lactase-phlorizin hydrolase, complex glycosides remain recalcitrant. Both in vitro fermentation models and in vivo animal studies demonstrate that specific microbial glycosidases, secreted by genera such as *Bifidobacterium* and *Lactobacillus*, are required to cleave these sugar moieties. This yields lipophilic aglycones ready for passive diffusion or further ring fission [117,118].

Following deglycosylation, the microbial enzymatic repertoire targets the core phenolic scaffolds. For flavonoids, this involves a multi-step pathway beginning with C-ring reduction and fission. Distinct bacterial species, such as *Flavonifractor plautii*, express flavone reductases that catalyse the hydrogenation of the C2=C3 double bond, a prerequisite for the subsequent ring cleavage that generates simpler phenolic catabolites [117].

#### 4.3.2. Convergent Pathways: Generation of Bioavailable Metabolites

Colonic biotransformation is characterised by the conversion of structurally diverse parent compounds into a convergent pool of low-molecular-weight metabolites with altered redox potential and enhanced bioavailability. (i) Ellagitannins and urolithins: High-molecular-weight ellagitannins (e.g., punicalagin) are hydrolysed by microbial tannases into ellagic acid. These precursors undergo sequential lactone ring cleavage and dehydroxylation by specialised consortia (e.g., *Gordonibacter* species) to yield urolithins (e.g., Urolithin A and B). Unlike their polymeric precursors, these metabolites exhibit significant lipophilicity and systemic circulation [115,119]. (ii) Catechins and phenolic acids: Galloylated catechins, such as epigallocatechin gallate, undergo ester hydrolysis to yield gallic acid and epigallocatechin (EGC). These are further metabolised via C-ring fission and dehydroxylation into valerolactones and simple phenolic acids (e.g., 3,4-dihydroxyphenylacetic acid). Recent evidence suggests that specific biotransformation pathways, analogous to those observed in fungal fermentation systems (e.g., *Eurotium cristatum*), are critical for generating metabolites with potent antioxidant retention [120]. (iii) Proanthocyanidins and NEPPs: NEPPs, often ignored in standard pharmacokinetic models, are depolymerized and metabolised into phenylvalerolactones and phenylpropionic acids. These metabolites represent the bioavailable currency of high-fibre, polyphenol-rich diets, linking colonic fermentation directly to systemic antioxidant status [114,116].

#### 4.3.3. Systems-Level Implications: Physicochemical Optimisation and Interindividual Variability

The pharmacokinetic relevance of these transformations is profound. By transforming polymers > 1000 Da into metabolites < 300 Da, the microbiota effectively bypasses the solubility–permeability limitations of the upper GI tract [118]. Furthermore, this “molecular simplification” often enhances blood–brain barrier penetrability. For instance, metabolites like urolithins have been identified in brain tissue, suggesting a direct link between colonic metabolism and neuroprotection [119]. At this stage, it becomes evident that the effectiveness of this “bioavailability rescue” is highly individualised. It emerges from a complex crosstalk between: (i) interactions within the food matrix (polyphenol–protein–polysaccharide binding); (ii) interactions between the matrix and the host along the oral-GI tract (involving saliva, enzymes, pH, bile, mucosa, microbiota); and (iii) the broader dietary context and food-choice patterns that continuously reshape these processes.

The concept of “metabotypes”—distinct phenotypes based on the capacity to produce specific metabolites like equol or urolithins—underscores the reliance on specific microbial guilds [115]. For example, the conversion of isoflavones to equol requires a specific reductive pathway present in only 30–50% of the population, creating a dichotomy in therapeutic efficacy [118].

In summary, the bioavailability of dietary polyphenols is not an intrinsic molecular property. Rather, it is the outcome of a sequential interaction between the food matrix and the biological processing environment. Table 3 consolidates these integrated sensory and physiological outcomes across the digestive tract.

**Figure 3:**
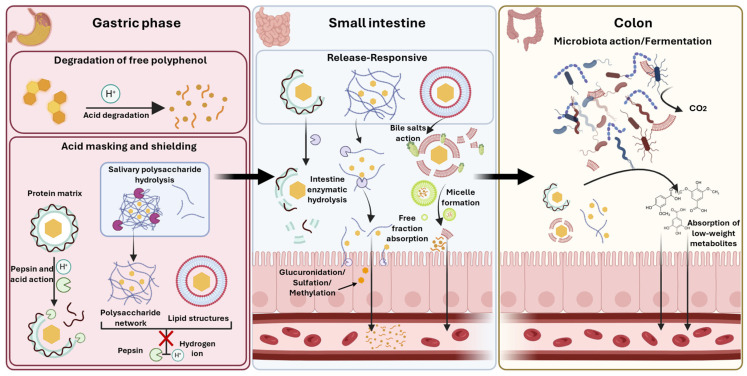
Modulation of dietary polyphenol stability and absorption across the gastrointestinal tract. In the gastric phase, protective molecular matrices (e.g., proteins, polysaccharides, and lipids) shield labile polyphenols from acid and enzymatic degradation. Upon reaching the small intestine, enzymatic hydrolysis and bile salts trigger matrix dissociation, releasing the free polyphenol fraction for epithelial absorption. Subsequently, these absorbed polyphenols undergo extensive Phase II first-pass metabolism (e.g., glucuronidation, sulfation, and methylation) within the enterocytes, which significantly alters the profile of the circulating systemic metabolites. Finally, unreleased or highly stable complexes proceed to the colon, where microbial fermentation dismantles the remaining physical barriers, converting complex parent polyphenols into bioavailable, low-molecular-weight metabolites. Created in BioRender. Ferreira, I. (2026) https://BioRender.com/50u6wux, accessed on 18 March 2026.

In fact, the pharmacokinetic trajectory begins indirectly with food matrices interactions (Section 2) and interactions inside the oral cavity (Section 3). It proceeds to gastric shielding (Section 4.1), where hydrophobic sequestration and steric hindrance protect labile compounds from acid degradation. Upon reaching the intestinal bottleneck (Section 4.2), physicochemical constraints dictate that only the free, unencapsulated fraction can be absorbed. Finally, the colonic rescue (Section 4.3) acts as the ultimate compensatory mechanism, dismantling the remaining matrix to liberate entrapped polyphenols. This stage effectively converts “locked” antioxidant potential into a systemic supply of bioactive phenolic acids and lactones [114,117]. Consequently, the microbiota does not merely degrade dietary compounds but actively upgrades their pharmacokinetic profile. Functional food design must therefore orchestrate a precise physicochemical balance. It must provide sufficient matrix strength to ensure sensory acceptance and gastric survival while simultaneously guaranteeing that “release-responsive” mechanisms—whether enzymatic, pH-dependent, or microbial—are effectively triggered to facilitate systemic absorption (Figure 3).

### 4.4. Beyond Bioavailability: Extraoral Bitter Taste Receptors as Targets of Dietary Polyphenols

Dietary polyphenols, particularly flavanols and tannins, that often carry bitter or astringent sensory profiles, can engage extraoral TAS2Rs across multiple tissues, triggering physiological responses independent of systemic absorption. This dual role positions TAS2Rs as molecular bridges between the challenging sensory properties of functional foods and their health potential, shifting focus from classical bioavailability (plasma levels of absorbed parent compounds) to receptor-mediated luminal signalling [121,122].

#### 4.4.1. TAS2Rs in the GI Tract: Hormone and Barrier Regulation

**Figure 4:**
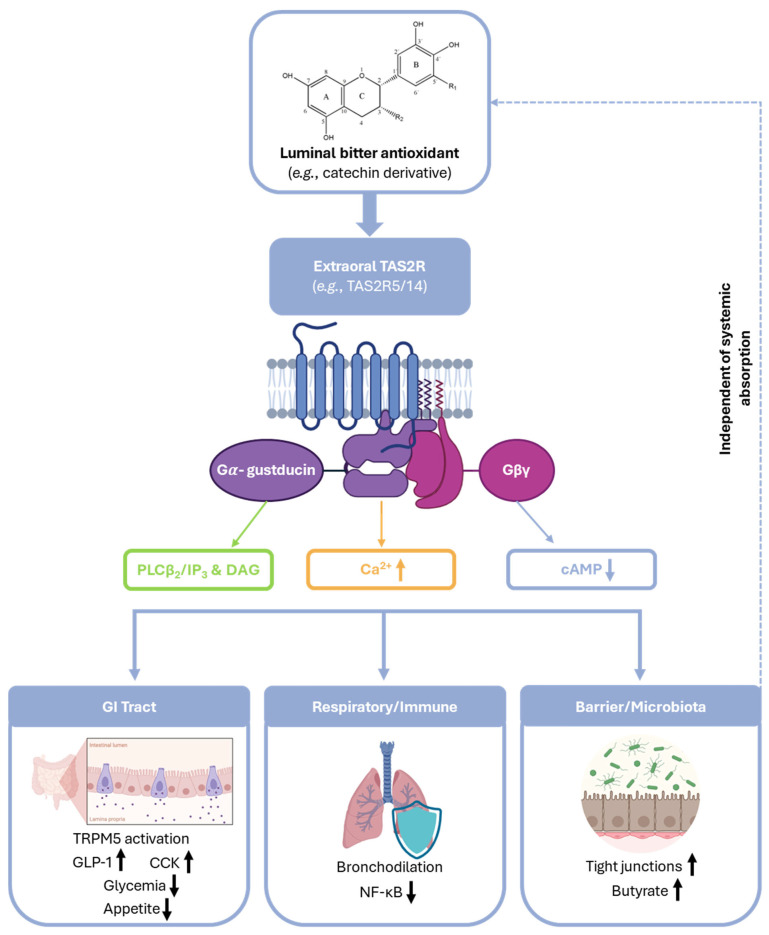
Activation of extraoral TAS2Rs by bitter polyphenols. Ligand binding triggers G-protein signalling (mediated by Gα: gustducin/Gβγ), converging on an elevation of intracellular Ca^2+^. This calcium efflux drives tissue-specific responses, including gut hormone secretion (GLP-1/CCK), bronchodilation, and barrier/microbiota modulation. This luminal receptor sensing occurs independently of classical systemic bioavailability. Symbols: ↑, increased; ↓, decreased.Created in BioRender. Ferreira, I. (2026) https://BioRender.com/43ap8xd, accessed on 18 March 2026.

TAS2Rs show broad expression along the human GI tract, from oesophagus to the colon, with the highest density found in colonic enteroendocrine cells and crypts. Over 20 receptors (e.g., TAS2R4, TAS2R5, TAS2R14) form a distributed “sensor array” that detects luminal bitter molecules from diet, microbiota metabolites, and host xenobiotics. Activation typically couples to Gα-gustducin, elevating intracellular Ca^2+^ and cyclic adenosine monophosphate (cAMP)ytoskeletal changes (Figure 4) [123,124,125].

A key example involves enteroendocrine hormone regulation. In human Hutu-80 intestinal cells, TAS2R14 agonism induces glucagon-like peptide-1 (GLP-1) secretion, while TAS2R5 activation boosts both GLP-1 and peptide YY (PYY)—critical hormones that suppress appetite, slow gastric emptying, and enhance insulin sensitivity. Crucially, such physiological responses occur at micromolar concentrations matching luminal polyphenol levels after realistic dietary intakes (e.g., 100–300 mg from cocoa or grape products). Importantly, these effective micromolar concentrations are readily achievable in the GI lumen, standing in stark contrast to the much lower, often nanomolar, and potentially insufficient concentrations that reach systemic circulation. Similarly, denatonium (a broad TAS2R agonist) stimulates cholecystokinin (CCK) release in STC-1 cells, delaying gastric emptying and altering nutrient transit—patterns replicated by phenolic extracts [124,126].

The grape seed proanthocyanidin extract (GSPE) study in aged rats provides compelling in vivo evidence. A 10-day oral GSPE dose (rich in bitter procyanidins) persistently altered Tas2r transcript profiles across stomach, jejunum, ileum, and colon. Remarkably, this effect was observed even 75 days post-dosing, long after clearance of parent flavanols. Network analysis revealed Tas2rs as high-betweenness hubs, linking upregulated barrier genes (e.g., tight junctions), reduced inflammatory markers (interleukin-6 (IL-6), tumour necrosis factor alpha (TNF-α)), shifted enterohormone patterns (elevated GLP-1), and enriched butyrate-producing microbiota. This cascade demonstrates how transient bitter polyphenol exposure can “imprint” durable GI adaptations that are relevant to ageing, obesity prevention, and metabolic health [127].

#### 4.4.2. Respiratory and Immune Defence via TAS2Rs

TAS2Rs extend to airway epithelia and smooth muscle, where they enhance host defence. In human primary ciliated bronchial cells cultured in vitro, bitter agonists like thioxane increase ciliary beat frequency and nitric oxide (NO) production, accelerating mucociliary clearance of pathogens. Furthermore, ex vivo studies on human and animal airway smooth muscle show that TAS2Rs (e.g., TAS2R10, TAS2R14) induce potent bronchodilation. This occurs via Ca^2+^-dependent K^+^ channel opening and remains effective even in asthmatic tissue desensitised to β2-agonists [128,129,130,131].

Dietary relevance emerges from shared pharmacology. Polyphenols activate the same subtypes (TAS2R10/14) as respiratory agonists, at concentrations achievable via circulation or local exposure. For instance, both denatonium and polyphenols relax guinea pig trachea. This hints that polyphenol-rich diets (e.g., tea, berries) might support lung function beyond their direct antioxidant effects [132,133].

In immune cells, TAS2Rs modulate inflammation. Neutrophils and macrophages express multiple subtypes. For example, salicin (phenolic glycoside from willow bark) activates TAS2R16 in gingival fibroblasts to suppress nuclear factor kappa B (NF-κB), thereby curbing IL-6/8 release. This aligns with GI anti-inflammatory data, suggesting that bitter polyphenols act as “immunosignals” at barrier interfaces [121,134].

#### 4.4.3. Metabolic Tissues and Systemic Reach

To explicitly delineate the current level of scientific confidence regarding extraoral sensing, it is crucial to distinguish between well-supported physiological effects and hypothesis-generating observations. While the role of extraoral TASR2 in GI hormone secretion (GLP-1/CCK) and airway bronchodilation is currently well-supported by robust ex vivo and in vivo animal models, their systemic reach requires careful interpretation. TAS2R expression appears in adipocytes, pancreatic β-cells, and vascular endothelium, opening metabolic avenues. In murine 3T3-L1 adipocytes, TAS2R activation alters lipolysis and leptin secretion, while islet TAS2Rs may fine-tune insulin release. Vascular TAS2Rs (e.g., TAS2R4/14) inhibit endothelial inflammation via the cAMP/PKA pathway. This potentially counters oxidative stress, providing a direct link to antioxidant themes [121,135,136]. Specifically, the activation of TAS2Rs exerts potent anti-inflammatory effects through a dual mechanism. It inhibits the NF-κB/NLRP3 axis while promoting NrfNLR family pyrin domain containing 3 (2 nuc)lear translocation, ennuclear factor erythroid 2-related factor 2 (hanc)ing cellular antioxidant capacity [136]. By coupling these pathways, extraoral TAS2Rs serve as redox-sensitive gatekeepers that translate chemosensory input into systemic protection against metabolic insults.

While direct polyphenol-TAS2R metabolism studies remain sparse, in vitro data (e.g., EGCG on TAS2R7/10) and GSPE’s systemic metabolic imprint support the concept. However, until further validated by human clinical trials, the direct role of polyphenol-TAS2R interactions in systemic metabolic regulation (e.g., modulating adipocyte lipolysis or pancreatic insulin release) remains largely hypothesis-generating, relying predominantly on in vivo evidence. Bitter phytochemicals thus function as distributed chemical signals, not mere scavengers [127,137,138].

To integrate the emerging evidence linking TAS2R activation and redox regulation, we compiled the most potent antioxidant polyphenols identified in Table 4. This table summarises their reported TAS2R interactions and antioxidant mechanisms. Most compounds were originally characterised as TAS2R agonists using heterologous expression systems (commonly HEK293T cells expressing individual human TAS2Rs) coupled to Ca^2+^ mobilisation assays [139,140,141]. Canonically, TAS2R activation triggers a G protein-mediated signalling cascade involving PLCβ2 activation, IP_3_ productiphospholipase C-β2 (on, a)nd intracelluinositol 1,4,5-trisphosphate (lar) Ca^2+^ release [142]. Beyond chemosensory perception, several of these polyphenols exhibit robust antioxidant activity. They achieve this either through direct radical scavenging or via modulation of endogenous defence pathways, particularly the Nrf2/ARE axis [143,144,145]. Notably, flavanols (e.g., EGCG), flavonols (e.g., quercetin), ellagitannins (e.g., punicalagin), and anthocyanins demonstrate both TAS2R activation and redox-regulatory properties. This suggests a potential mechanistic convergence between bitter chemosensation and cellular antioxidant signalling [139,141]. The integration of TAS2R-mediated Ca^2+^ signalling with Nrf2-dependent antioxidant responses may represent a previously underappreciated link between dietary polyphenols and extraoral TAS2R physiological functions.

Beyond mechanistic considerations, these findings have important implications for how polyphenol bioactivity is interpreted in nutritional science and food design.

#### 4.4.4. Implication for Bioavailability and Food Design

Traditional bioavailability metrics undervalue bitter polyphenols. While these compounds typically exhibit < 5% absorption, their luminal TAS2R activation yields robust hormone, barrier, and microbiota effects. Consequently, the concept of “signal bioavailability”—defined by receptor engagement duration and downstream reprogramming—better captures their systemic impact [127,137,138].

However, formulation strategies must balance sensory acceptability with preservation of biological activity. Excessive bitterness masking that severely reduces luminal bioaccessibility/bioavailability may blunt these TAS2R-mediated benefits. Importantly, masking approaches must preserve a releasable pool of intact, receptor-active polyphenols in the GI tract rather than causing irreversible sequestration or degradation. Instead, advanced formulation strategies must (i) employ smart encapsulation (e.g., enteric coatings or the pH-responsive hydrogels discussed in Section 4.1.2), which physically sequesters polyphenols to prevent premature oral receptor activation while ensuring triggered dissociation in the lower GI tract where TAS2R expression is dense; (ii) use fermentation processes that enrich TAS2R-active phenolic fractions; and (iii) co-formulate with milder bitter agonists to flatten the sensory peak of bitterness to tolerable levels while sustaining cumulative receptor signalling to trigger satiation hormones [138].

This “functional food paradox” is experimentally demonstrated in specific polyphenol-rich foods. Green tea infusions, dominated by bitter TAS2R39 agonist EGCG, often cause oral aversion at higher doses yet stimulate intestinal TAS2R39 to induce GLP-1 secretion and Nrf2-mediated antioxidant defences [143,146]. Similarly, highly astringent GSPE limit sensory acceptance in fortified foods, but these same polymeric procyanidins persistently stimulate intestinal TAS2Rs, upregulating tight junction proteins and modulating the gut–brain axis in animal models [127]. Dark chocolate provides another example, where increased polyphenol bitterness reduces consumer liking, yet higher cocoa content correlates with improved metabolic biomarkers.

Ultimately, extraoral TAS2Rs explain a key paradox: the same bitterness that limits palatability triggers health benefits in gut issues [135].

### 4.5. Toxicological and Anti-Nutritional Implications of Macromolecular Binding and Redox Cycling

While the health-promoting properties of dietary polyphenols are widely documented, the specific structural features driving their bioactivity can concurrently pose toxicological and anti-nutritional risks [179,180]. Dietary polyphenols, particularly high-molecular-weight tannins and complex flavonoids, possess extended aromatic networks and dense hydroxylation patterns. These structural characteristics facilitate avid non-covalent binding, primarily via extensive π-π stacking and hydrophobic interactions, which can severely disrupt regular biochemical processes [181].

From an anti-nutritional perspective, polyphenols act as potent metal chelators. In the gastrointestinal lumen, the chelation of transition metals—most notably non-haem iron—significantly impairs their absorption. This can exacerbate iron deficiency in vulnerable populations [179,180]. Interestingly, the secretion of PRPs in human saliva may act as an adaptive defence mechanism. These salivary proteins bind to tannins with high affinity, potentially mitigating the intestinal chelation of iron. However, the anti-nutritional risk persists with high-dose consumption [182]. Furthermore, the robust affinity of these large hydrophobic aromatic systems for functional proteins actively interferes with host digestion. The structural aggregation with proteins, namely digestive enzymes, can inhibit their enzymatic activity, thereby diminishing the bioaccessibility of essential macronutrients.

At the systemic and cellular membrane levels, the biological interactions of these large polyphenolic structures raise explicit toxicological concerns. High polyphenol consumption can inhibit key drug-metabolising enzymes, such as cytochrome P450 (e.g., CYP3A4), alongside apical efflux transporters like P-gp and MRP2 on the intestinal epithelium [181]. This inhibition compromises vital extrusion pathways responsible for clearing xenobiotics and drugs, resulting in potential adverse effects and toxic interactions [181]. Additionally, specific compounds, such as certain isoflavones, can act as endocrine disruptors. They inhibit enzymes involved in thyroid hormone biosynthesis, thus presenting goitrogenic effects [180].

Finally, the redox chemistry of polyphenols is highly context-dependent. Under specific conditions—such as high-dose supplementation, alkaline pH, or the presence of transition metal ions (e.g., Fe^3+^, Cu^2+^)—polyphenols can undergo auto-oxidation and initiate detrimental redox cycling [183,184]. This transition converts them from radical scavengers into potent pro-oxidant molecules, generating ROS through Fenton-type reactions. These pro-oxidative states can deplete cellular antioxidant defences, induce severe oxidative stress, and provoke DNA damage with potential genotoxic and mutagenic effects [184].

Ultimately, these disruptive biochemical interactions provide a clear physiological rationale for the innate psychological barriers discussed later in this review. The human sensory aversion to highly bitter and astringent polyphenol-rich matrices likely functions as an evolutionary, psychobiological defence mechanism. This aversion protects the host against the potential systemic toxicity and anti-nutritional properties of these large aromatic structures [143,146].

## 5. Driving Consumer Preferences: Physiological and Sensory Perspectives

The translation of molecular architecture and oral processing into long-term dietary patterns is an essential phase in the functional food chain. In Section 4 we demonstrated how the food matrix actively protects bioactive compounds during gastric digestion. However, this physiological advantage is ineffective if the food is never swallowed. The molecular interactions described in Section 2 and the oral biochemistry detailed in Section 3 determine the physicochemical state of the bolus. Yet consumer acceptance represents a higher-level integration. It combines these biochemical inputs with genetic predispositions, psychological traits, and post-ingestive feedback. Recent studies on sustainable plant-based ingredients [185] have shown that the “sensory cost” of adding polyphenols—mostly manifested as bitterness and astringency—act as a significant biological barrier to intake. This barrier is not merely a matter of hedonic preference but involves complex physiological signalling that cannot be entirely overcome by health claims alone. Consequently, developing successful functional foods requires a deep understanding of how sensory perception evolves dynamically during consumption.

### 5.1. Sensory Barriers to Consumption

A significant limitation in predicting consumer acceptance lies in the reliance on static sensory profiling. This traditional approach often fails to capture the dynamic nature of eating behaviour and the psychological barriers to novel foods. In reality, the rejection of polyphenol-rich foods is driven by the interplay between the dynamic sensory profile of the matrix and the psychological traits of the consumer. Using Temporal Dominance of Sensations (TDS) in human sensory panels, Silva et al. [186] demonstrated that astringency and bitterness exhibit a cumulative “carry-over” effect. In polyphenol-rich matrices, the lubricating salivary film is gradually depleted with each successive sip. This results in a “sensory build-up” where dryness and roughness eventually outweigh positive attributes. Ultimately, this causes palate fatigue and triggers negative emotional responses (such as dissatisfaction), leading to rejection before an entire portion is consumed.

However, this sensory input is inevitably filtered through the consumer’s personality traits, which can act as powerful psychological barriers. De Toffoli et al. [187] presented evidence that individual differences in the acceptance of polyphenol-rich foods are driven by two key psychological indices: Food Neophobia (FN) and General Sensitivity to Punishment (SP). Food Neophobia, measured by the Food Neophobia Scale, reflects a reluctance to eat new foods. It act as an evolutionary defence against possible toxicity. Individuals with higher FN scores systematically reject the complex sensory profiles of antioxidant-rich foods due to a lack of familiarity, interpreting novel bitterness as a danger signal. At the same time, SP, derived from the Reinforcement Sensitivity Theory, measures the responsiveness of the Behavioural Inhibition System (BIS) to aversive stimuli. For high-SP consumers, the bitterness of polyphenols is physiologically decoded as a “sensory punishment”. This results in increased avoidance learning and a strong reaction to the negative affect of off-flavours.

In addition to these psychological barriers, Spinelli et al. [188] identified “Sweet Liking Phenotypes” as a third critical factor of acceptance. Their research stratifies consumers based on their optimal sweetness preference, revealing a distinct behavioural pattern. Specifically, “High Sweet Likers” display a strong negative correlation between liking and bitterness. Consequently, they require significant sugar masking to accept functional foods. In contrast, consumers with an “Inverted-U Shaped” phenotype (who favour moderate sweetness levels) exhibit a significantly higher baseline acceptance of polyphenol-rich vegetables and beverages without added sugar. This suggests that the rejection of functional foods is not uniform. Rather, it is concentrated within specific phenotypic groups (High Sweet Likers/High Neophobia) that demand targeted formulation strategies. Conversely, Inverted-U phenotypes represent a naturally compliant market segment for polyphenol-rich diets. These studies indicate that such psychobiological characteristics are often stronger predictors of food choice than biological taste sensitivity alone. Thus, predictable rejection phenotypes are produced by the interaction of stable psychological traits with dynamic oral events.

### 5.2. Genotype and Perception

Beyond the psychological filters of neophobia, the “bioavailability of flavour” is significantly influenced by biological individuality, which creates a genotype–phenotype interaction that stratifies the population. This stratification is primarily driven by Single Nucleotide Polymorphisms (SNPs)—variations at a single position in a DNA sequence that controls the functionality of taste receptors and oral physiology. The “average consumer” is a statistical fallacy. In reality, intake is governed by these specific polymorphisms, most notably in the TAS2R38 gene. Three specific missense SNPs (rs713598, rs1726866, and rs10246939) result in amino acid substitutions that create the distinct PAV (Proline-Alanine-Valine) or AVI (Alanine-Valine-Isoleucine) haplotypes. Nor et al. [189] established that individuals possessing the PAV/PAV diplotype (“supertasters”) perceive the bitterness of turnips and other polyphenol-rich crops at intensities much higher than “non-tasters” (AVI/AVI), regardless of the cooking method employed. Similarly to this, Sandell and Breslin [190] demonstrated that this genetic sensitivity functions as a hard biological barrier: individuals with the sensitive PAV haplotype experience significantly higher levels of bitterness from polyphenol-rich vegetables, resulting in lower acceptance and intake. For these phenotypes, the “unmasking” of polyphenols may trigger a stronger rejection response that could be difficult to overcome through formulation strategies alone. Moreover, polymorphisms in the *CA6* gene (specifically rs2274333), which codes for the salivary trophic factor gustin, have been linked to fungiform papillae density. Variations at this locus can increase tactile and chemesthetic sensitivity, further modulating the perception of astringency and texture in functional matrices [191,192].

However, receptor sensitivity is not the sole determinant of intake; it is also modulated by the individual’s salivary profile. While Section 3 discusses how saliva impacts chemical stability, from a sensory perspective, the proteomic composition determines the intensity of the “drying” sensation. The work of our group [96] has been pivotal in elucidating the molecular hierarchy of this interaction. These authors showed that aPRPs act as the primary, high-affinity targets for condensed tannins, forming stable and typically insoluble aggregates. A complementary longitudinal study of the same research group [193], tracking 17 volunteers over one year reported that, although individual salivary profiles vary over time, total salivary protein levels remain relatively stable. This reinforces the concept that specific protein composition, rather than bulk protein concentration, drives inter-individual differences in astringency. Using procyanidin mixtures, the authors observed a highly selective depletion of specific proteins, particularly statherin and P-B peptides, across participants, largely independent of baseline saliva phenotype. Sensory data further suggested that individuals with higher levels of certain salivary proteins may be more sensitive to astringent “drying”. Furthermore, the specific composition of the astringent stimulus (in this case a procyanidin structure/mixture) can be more determinant of these interactions than total protein availability. Additionally, in a very recent large-scale study performing whole-genome sequencing analyses of saliva (>12,000 participants) provided crucial insights. Kamitaki et al. [194] demonstrated that host genetic variation and microbial genomic adaptation jointly shape oral community structure and oral health risk. Specifically, variations at loci involved in salivary and mucosal ecology, including AMY1 (salivary amylase copy-nu*mber* and coding variants) and glycosylation-related genes such as FUT2 and ABO, associates with *both* the *abu*ndance profiles and strain-level gene content of resident oral taxa. This is consistent with genetically defined niches that select distinct microbial functions. Notably, AMY1 copy number and rare miss*ense* variants were linked to an increased likelihood of tooth loss or denture use. Concurrently, multiple FUT2/ABO-dependent signals supported host–microbe co-adaptation, including bacterial genes predicted to catabolise or bind host histo-blood group antigens and adhesin repertoires enriched in “secretor” backgrounds. Together, these findings imply that commensals can exploit human glycan diversity for nutrition and colonisation.

However, it remains unclear how microbiome-driven flavour modification related to long-term eating habits. Importantly, the implications of this individualised ecosystem extend beyond oral health to sensory perception. Distinct from gut metabolic activities, the bacterial communities on the tongue dorsum contribute directly to the “in-mouth” flavour generation as discussed in Section 3. According to Schwartz et al. [56], specific oral microbiotypes possess distinct glycosidase activities capable of hydrolysing glycosylated aroma precursors in situ. This generates personalised aroma release patterns that differ between individuals consuming the exact same matrix. Such variation explains heterogeneity in consumer liking based on the specific “flavour bouquet” released during mastication.

This physiological landscape is far from static; it evolves significantly with ageing, introducing a paradox in the acceptance of functional foods by the elderly population. While the detection threshold for astringency rises with age (i.e., seniors are less sensitive to the sensation), Wang et al. [195] showed that this “advantage” is often counterbalanced by a reduction in salivary flow. Once the threshold is crossed, the lubricating salivary film is depleted, making mechanical friction more damaging and persistent. Moreover, this landscape is modulated by gender-specific effects. Large-scale population studies indicate that women generally possess a higher fungiform papillae density and stronger bitter taste responsiveness than men. This may render them more susceptible to the “sensory cost” of polyphenol fortification [196]. Additionally, nutritional status, specifically Body Mass Index (BMI), appears to reshape sensory physiology. According to studies by Proserpio et al. [197] and Archer et al. [198], obesity is associated with a chronic low-grade inflammation that can reduce the abundance of fungiform papillae and change the expression of taste genes. Consequently, this creates a “blunted” sensory landscape where higher concentrations of stimuli are required to elicit the same hedonic reward. This potentially complicates the acceptance of subtle-flavoured functional foods.

### 5.3. Cognitive and Physiological Interaction

In the end, integrating functional foods into a regular diet requires resolving the conflict between the innate sensory aversion (described in Section 5.1) and the biological or cognitive drive to consume. While Section 4 detailed the physiological mechanisms of extraoral sensing, this section addresses how those signals are integrated by the brain to modulate the hedonic response.

As referred throughout the text, the primary flavour properties linked to polyphenols are typically bitterness and astringency. Crucially, these are not merely peripheral chemical events, but centrally integrated percepts. Gustatory and oral somatosensory (trigeminal and texture) signals converge in the anterior insula and frontal operculum, which comprise the primary taste cortex. These signals are subsequently transformed within the orbitofrontal and cingulate networks. Here, they encode affective value, attention, and action tendencies towards foods. Neuroimaging indicates that astringent stimuli (e.g., tannic acid) and bitter stimuli activate overlapping yet partially segregated insular subregions. Simultaneously, they engage orbitofrontal and cingulate cortices. This supports the idea that “antioxidants-rich” sensations (often carried by polyphenols) are represented as integrated flavour–mouthfeel objects with an aversive/alerting component [199]. Mechanistically, polyphenols can drive this integration by activating bitter GPCR pathways, such as TAS2Rs. In certain cases, TRP-mediated trigeminal signalling, strengthening the coupling between taste and irritation/drying cues. Importantly, inter-individual variability may arise from central processing differences, rather than solely from salivary proteins or receptor genetics. For instance, PROP-sensitive individuals sh 6-*n*-propylthiouracilo(w al)tered prefrontal responses during bitter perception. Furthermore, neural responses shift depending on evaluative task demands. This implicates cognition, learning, and valuation in explaining that the same polyphenol load is “pleasantly complex” for some and “overly bitter/astringent” for others [200].

Recent evidence supports a specific role for prefrontal circuitry in shaping astringency-related food responses. Using sensory profiling combined with functional near-infrared spectroscopy (fNIRS), Kew et al. [201] demonstrated that isolated plant proteins (with tannic acid as an astringent comparator) elicit a distinct haemodynamic signature in the dorsolateral prefrontal cortex (DLPFC). Specifically, activation in the right DLPFC (BA9) tracks astringency intensity, even when taste and viscosity are controlled. Conversely, left DLPFC responses align more consistently with hedonic evaluation (liking/disliking). Importantly, their parallel cellular assays demonstrated that the same samples driving stronger DLPFC responses also caused greater salivary/mucin depletion on oral-epithelium models. This provides a direct link between oral biophysics and neural coding. Collectively, these findings reinforce the hypothesis that inter-individual differences in “antioxidant-rich” bitterness/astringency acceptance arise from multiple levels. They stem not only from peripheral receptor and saliva phenotypes but also from variability in the prefrontal processing of intensity, after-feel persistence, and learned negative or positive value. Ultimately, this central integration governs food choice and repeat intake.

**Figure 5:**
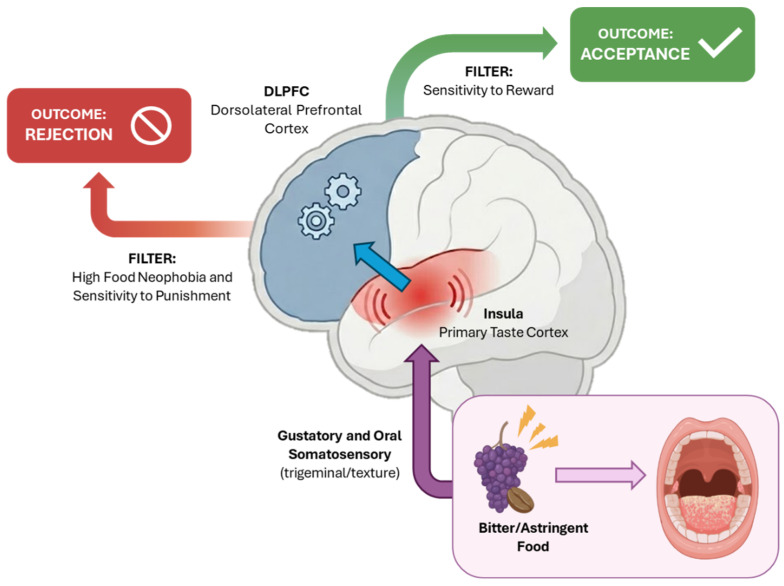
Psychobiological decision tree illustrating how specific consumer traits filter the bitter/astringent sensory input of functional foods, leading to diverging pathways of rejection (safety seeking) or acceptance (natural tolerance versus reward-driven conditioning). Created in BioRender. Ferreira, I. (2026) https://BioRender.com/pq41laq, accessed on 18 March 2026.

This neural calculation of “value” is intrinsically modulated by individual personality profiles. These profiles ultimately determine whether an aversive sensory signal is amplified or suppressed. Within this psychobiological framework, De Toffoli et al. [187] highlighted the role of specific psychological traits. While FN and SP act as barriers, Sensitivity to Reward (SR)—linked to the Behavioural Activation System (BAS)—functions as a crucial driver for intake. Consumers with high SR scores are psychologically motivated to override the immediate sensory “punishment” of bitterness in anticipation of a neurobiological reward (e.g., the psychostimulant effects of methylxanthines or the perceived health benefits). For these individuals, the decision to consume is not driven by the sensory profile itself, but by the dopaminergic anticipation of the post-ingestive consequence, effectively suppressing the avoidance/intake signal (Figure 5).

Where internal psychological drives prove insufficient, external cognitive cues, or “credence attributes,” play a pivotal role. They modulate the sensory tolerance threshold by offering an alternative form of value. In the context of the global transition towards plant-based diets, Michel et al. [202] provided empirical evidence that consumers are willing to engage in a conscious “sensory trade-off”. Their experimental data revealed that when products are strongly associated with environmental sustainability, positive cognitive associations can buffer against the negative impact of off-flavours typical of plant matrices. This suggests that the “Sustainability Halo” acts as a cognitive filter, raising the rejection threshold for bitterness and astringency, provided the off-notes do not exceed a critical intensity where the biological aversion becomes challenging.

However, cognitive motivation alone may be transient. Enduring dietary shifts require biological reinforcement through Flavour-Nutrient Learning. In this Pavlovian framework, the metabolic consequences of digestion served as the “unconditioned stimulus”. Specifically, this involves the extraoral *TAS2R* activation and subsequent release of satiety hormones (CCK, GLP-1) described in Section 4. The brain eventually maps the specific sensory signature of the polyphenol (the “conditioned stimulus”) onto these positive metabolic feedback loops. Duffy et al. [203] observed this phenomenon empirically, noting that repeated exposure to polyphenol-rich Aronia berry juice significantly increased hedonic ratings over time. This change was not merely psychological habituation (i.e., sensory fatigue) but a physiologically reinforced preference. This mechanism is paradigmatically illustrated by coffee consumption. Despite its innate aversiveness, coffee acts as a potent vehicle for caffeine-mediated reinforcement. In a large-scale Mendelian randomisation study involving human cohorts, Ong et al. [204] revealed a distinct dichotomy in bitter perception. Individuals with higher genetic sensitivity to PROP or quinine consumed less coffee, which is consistent with innate aversion. Conversely, those with higher sensitivity specifically to caffeine consumed significantly more. For these consumers, the specific bitter signal of caffeine functions not as a deterrent, but as a reliable conditioned stimulus. It predicts the psychostimulant reward (e.g., adenosine blockade, alertness), thereby reinforcing the acquisition of a liking for the sensory profile.

Over time, the “bitter” taste may shift from functioning as a warning signal to serving as a learned predictor of metabolic reward. This explains how functional foods can transition from being simply “tolerated for health” to being “hedonically preferred” in the long term.

Table 5 summarises these multi-level determinants, highlighting the complex interplay between biological markers and psychological traits in shaping consumer acceptance.

### 5.4. Bridging Molecular Design and Consumer Behaviour: A “Sensory-by-Design” Framework

The integration of the chemical and psychobiological levels described above suggests that the development of successful functional foods must move well beyond simple flavour masking. To fully bridge the gap between polyphenol chemistry (Section 2), oral biochemistry (Section 3), and consumer intake (Section 5), a “Sensory-by-Design” framework should be proposed. This practical approach leverages molecular interactions to systematically navigate the sensory barriers imposed by individual genotypes and psychological traits through a sequential process.

Specifically, this involves: (i) formulation choices: the process begins at the molecular level by selecting specific food matrix macromolecules (e.g., proteins or polysaccharides) to create non-covalent complexes or encapsulation systems with the target polyphenols [39,40,70]; (ii) expected sensory profile coupled to gastrointestinal release: these physicochemical interactions are purposefully designed to lower the free fraction of highly reactive bioactives in the oral cavity, which attenuates the intensity of bitterness and modifies oral lubrication to reduce astringency and later on can be released in small intestine to be bioaccessible and bioavailable [4,49]; and (iii) testing approach: rather than relying solely on static hedonic scores, sensory evaluation should employ dynamic profiling methods, such as TDS, to accurately capture the cumulative “carry-over” effect and the sensory build-up of astringency over repeated sips [176].

Furthermore, this framework requires: (iv) biomarkers and outcomes: sensory testing must be coupled with physiological validation. Relevant biomarkers include measuring the selective depletion of salivary proteins (e.g., statherin and aPRPs) to objectively quantify astringent interactions [183] or using fNIRS to track DLPFC activation as a neural signature of sensory aversion [191]. Crucially, formulations should also be screened for extraoral TAS2R activation to ensure that the “masked” polyphenol retains its luminal signalling capacity and successfully triggers metabolic benefits, such as GLP-1 or CCK release [120,122]; (v) consumer segmentation: the optimised formulation is matched to specific consumer phenotypes, recognising that groups like “supertasters” (PAV/PAV haplotypes), individuals with high FN, or “High Sweet Likers” possess fundamentally different tolerance thresholds [177,178,179]; and, finally, (vi) iteration: feedback from physiological biomarkers and segmented consumer acceptance dictates necessary adjustments to the initial formulation choices.

**Figure 6:**
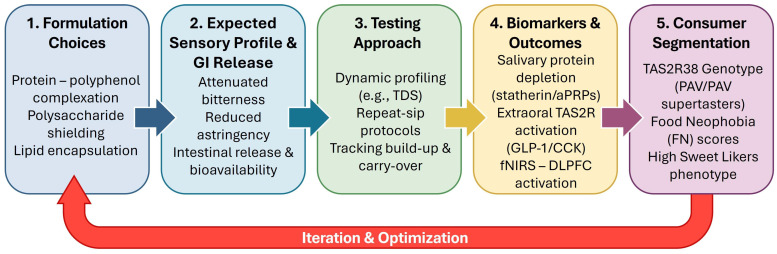
Proposed “Sensory-by-Design” framework for functional food development. This iterative model bridges molecular chemistry and consumer psychobiology by linking formulation choices directly to sensory profiles, physiological biomarkers, and targeted consumer segmentation.

By utilising this proposed framework (Figure 6), food developers can systematically transform the chemical protection provided by the food matrix into an optimised sensory experience, ensuring that polyphenol-rich foods are not only biologically active but also widely accepted by consumers.

## 6. Conclusions and Future Directions

Incorporating dietary antioxidant polyphenols into everyday foods is not simply a matter of “masking” unpleasant notes. In polyphenol-rich products, bitterness and astringency often reflect the same chemical features that drive bioactivity—namely, dense phenolic hydroxylation, redox capacity, and strong non-covalent binding to proteins and interfaces. In the oral cavity, polyphenols bind salivary PRPs and mucins, disrupt lubrication, and alter colloidal stability, producing astringency and bitterness. Those same interaction chemistries can also help explain why many polyphenols persist through upper GI digestion. They are extensively transformed by gut microbiota, exerting physiological effects via local signalling pathways rather than relying solely on high plasma concentrations.

This linkage motivates a vital shift from taste masking to a Sensory-by-Design” strategy in functional food engineering. Instead of removing polyphenols or compensating with sugar, fat or salt, formulators can use targeted biopolymer matrices (e.g., protein–polysaccharide complexes, emulsions, microgels) to control microenvironment and exposure. This approach effectively reduces the salivary contact that drives oral aversion. Simultaneously, it enables targeted release and transformation where physiological function is maximised, including the distal intestine.

Key frontiers now lie in closing the gap between chemistry, physiology, and eating behaviour. Clinically, extraoral TAS2R signalling should be validated with endpoints that capture receptor-mediated and local effects. This approach complements traditional plasma absorption metrics with the broader concept of “signal bioavailability” (e.g., tracking gut hormone responses, motility, inflammatory tone, and microbiome-derived metabolites). In parallel, neurophysiological tools such as fNIRS can help resolve how oral bitterness and astringency, post-ingestive feedback, and learned rewards shape acceptance over repeated exposures. Ultimately, progress will require personalization. This means aligning matrix design with genetic variation (e.g., TAS2R polymorphisms), oral physiology, microbiome profiles, and psychological traits such as FN to move beyond a flawed, one-size-fits-all approach.

## Figures and Tables

**Table 1:** Major dietary polyphenols: structural characteristics, sources, and structure–activity relationships (SAR). Adapted from [4].

Class	Subclass	Examples	Food Source	SAR	Key References
Non-flavonoids	Lignans	Enterolactone 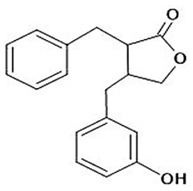	Linen seeds, Broccoli	**Moderate**: Direct scavenging is limited; health effects rely heavily on microbial conversion into enterolignans.	[8]
	Stilbenes	Resveratrol 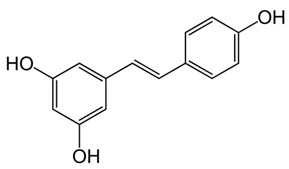	Red grapes, Wine	**Moderate-High:** The ethylene bridge linking two phenolic rings allows extended electron delocalization.	[9]
	Phenolic Acids	Hydroxibenzoic Acid 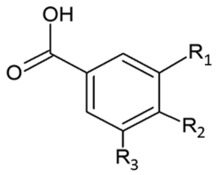 R_1_, R_2_ = OH; R_3_ = H: protocatechuic acid R_1_, R_2_, R_3_ = OH: gallic acid Hydroxicinnamic Acid	Coffee, Chocolate, Spinach	**High:** The presence of catechol or pyrogallol rings maximises electron donation and radical stabilisation.	[10,11]
		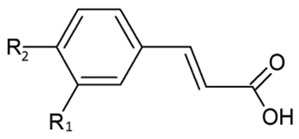 R_1_, R_2_ = OH: caffeic acid R_1_ = OMe; R_2_ = OH: ferulic acid			
Flavenoids	Anthocyanins	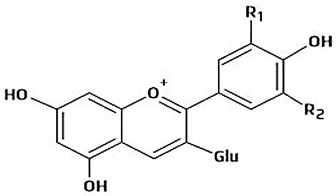 R_1_ = OH; R_2_ = H: cyanidin R_1_, R_2_ = OH: delphinidin R_1_, R_2_ = OMe: malvidin	Red fruits, berries	**High (pH-dependent):** Function as strong hydrogen donors (flavylium cations), but stability is strictly dictated by matrix pH.	[12]
	Chalcones	Xanthohumol 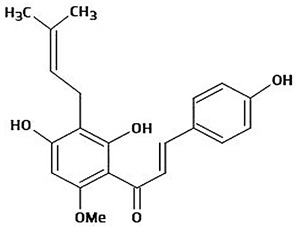	Beer	**Moderate:** Open-chain flavonoids where activity is modulated by the α,β-unsaturated carbonyl system.	[13]
	Flavan-3-ols	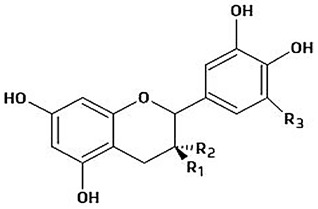 R_1_ = OH; R_2_, R_3_ = H: (+)-catechin R_1_, R_3_ = H; R_2_ = OH: (-)-epicatechin R_1_, R_2_, R_3_ = OH: (+)-gallocatechin	Red grapes, Wine	**Very High:** Dense hydroxylation (especially galloylation) drives exceptional scavenging and strong salivary protein precipitation.	[14]
	Flavonols	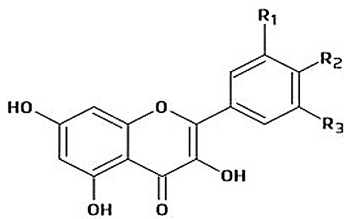 R_1_, R_2_ = OH; R_3_ = H: quercetin R_1_, R_2_, R_3_ = OH: myricetin	Black and Green Tea, Onions	**High:** Represent the optimal radical-scavenging template (C2=C3 double bond + 4-oxo group + 3-OH group).	[15,16]
	Flavones	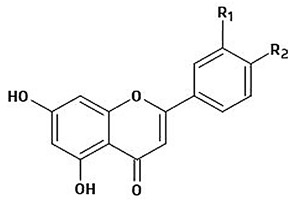 R_1_, R_2_ = OH: luteolin R_1_ = H; R_2_ = OH: apigenin	Carrot, Olive Oil, Peppers	**Moderate:** Structurally similar to flavonols but lack the crucial 3-OH group, which lowers their direct antioxidant power.	[17]
	Isoflavones	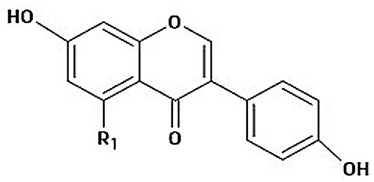 R_1_ = H: daidzein R_1_ = OH: genistein	Soy Sauce, Milk	**Low-Moderate:** B-ring attachment at C3 (instead of C2) disrupts extended conjugation, reducing direct reactive oxygen species (ROS) scavenging.	[18]

**Table 2:** Summary of the main enzyme classes present in human saliva.

Source	Functional Class	Example	Function	Key References
Human	Hydrolases/esterase-like	α-amylase Lingual lipase Carboxylesterases	Modifies starch structure and viscosity, indirectly impacting diffusion and release of flavour-active molecules; Cleavage of ester bonds in certain aroma precursors or lipid-derived substrates (even modest activity can matter under repeated exposure)	[51,52]
	Peptidases	Aminopeptidases Dipeptidyl peptidase-IV (DPP-IV)	Generally lower than in the gut, but relevant for modifying peptides and potentially altering the availability of binding partners in the salivary film	[53]
	Oxidoreductases & defence enzymes	Lysozyme Peroxidases Lactoferrin-related activities Aldo-keto reductase	Redox-active components can influence oxidative reactions at the food-saliva interface, which is particularly relevant for polyphenols due to their redox chemistry and propensity to form quinone-like intermediates under certain conditions	[54]
	Hydro-lyase	Carbonic anhydrase	Contributes to pH regulation and CO_2_/bicarbonate dynamics, indirectly shaping taste perception and oral chemistry	[55]
Microbial	Glycosidases	β-glucosidases	Hydrolyse glycosylated flavour precursors, releasing volatile aglycones and changing the retronasal aroma profile during chewing	[56]
	Esterases	Esterase	Hydrolyse dietary and aroma-active esters into alcohols and acids during chewing, reshaping the in-mouth volatile profile and perceived aroma intensity and flavour character	[57]
	Reductases	Diacetyl reductaseAlcohol dehydrogenase	Can reduce aroma-active carbonyls (aldehydes/ketones) and related compounds into different, often less pungent products (e.g., alcohols) during eating, shifting the in-mouth volatile profile and perceived flavour/aftertaste	[58,59,60]
	Decarboxylases	Ornithine decarboxylase	Convert amino acids (and some organic acids) into smaller, volatile/basic products (most notably biogenic amines) thereby shifting pH and generating odour/taste-active compounds that influence flavour and aftertaste	[61]

**Table 3:** Integration of sensory and physiological outcomes of polyphenol-rich functional foods across the digestive tract.

Phase/Location	Key Mechanisms	Sensory Consequences (In-Mouth) and Post ingestive	Physiological Consequences (Bioaccessibility and Biomarkers)
Food Matrix	Non-covalent binding of polyphenols to proteins and polysaccharides; encapsulation	Mitigation of off-flavours: reduces free polyphenols, masking bitterness and astringency	Gastric shielding: physically can protect labile polyphenols from degradation in the acidic environment
Oral Cavity	Salivary protein precipitation; mucosal pellicle interaction; enzymatic biotransformation	Flavour perception: drives astringency and bitterness; modulates aroma release profiles	Initial breakdown: prepares the matrix for digestion; generates transient complexes; impact on bioaccessibility/bioavailability
Stomach	High acid environment and proteolysis by pepsin; disruption of some aggregates	Post-ingestive signalling: delay of gastric emptying and generation of early satiety signals	Initial matrix breakdown (e.g., swelling and partial digestion); biopolymer network acts as a physical barrier, shielding labile polyphenols from acid-induced degradation
Small Intestine	Pancreatic enzymes and bile salts digest the matrix; luminal TAS2R sensing	Metabolic feedback: post-ingestive signalling modulates satiety via brain–gut axis	Micellization and absorption: release of the free fraction: ↑ GLP-1 and CCK secretion
Colon	Microbial metabolism: fermentation of not absorbed matrices and complex polyphenols	Post-ingestive signalling: modulation of the gut–brain axis and long-term satiety via short-chain fatty acid (SCFA) production	“Colonic Rescue”: biotransformation into highly bioavailable low-molecular-weight metabolites; prebiotic-like effect selectively enriching beneficial microbiota

**Table 4:** Antioxidant polyphenols with direct evidence of human TAS2R activation, considering only extraoral TAS2Rs and canonical TAS2R Ca^2+^ signalling.

Polyphenol Class/Compound	TAS2R/Site *	Reported Outcome	Evidence Type	References
**Flavanols**				
(-)-Epigallocatechin gallate (EGCG)	hTAS2R14, hTAS2R39 † (GI tract incl. small intestine/colon, respiratory epithelium)	Pathway: TAS2R → PLCβ2/IP_3_ → Ca^2+^ ↑; Nrf2/ARE Effect: ↑ HO-1/NQO1; ↓ ROS/lipid peroxidation	TAS2R in vitro; antiox in vivo/in vitro	[139,143,146]
(-)-Epicatechin gallate (ECG)	hTAS2R14, hTAS2R39 (GI tract, respiratory)	Pathway: TAS2R → PLCβ2/IP_3_ → Ca^2+^ ↑; redox modulation Effect: ↓ oxidative injury markers (↓ MDA; ↑ SOD)	TAS2R in vitro; antiox in vivo/in vitro	[147,148]
(-)-Epicatechin (EC)	hTAS2R39 (GI tract, vascular)	Pathway: TAS2R → PLCβ2/IP_3_ → Ca^2+^ ↑; antioxidant defence Effect: ↓ ROS/MDA; ↑ SOD/GSH	TAS2R in vitro; antiox in vivo	[139,144]
(+)-Catechin	hTAS2R14, hTAS2R39 (GI tract, vascular)	Pathway: TAS2R → PLCβ2/IP_3_ → Ca^2+^ ↑; Nrf2/HO-1 Effect: ↓ GI oxidative damage; ↑ HO-1 (Nrf2-linked)	TAS2R in vitro; antiox in vivo	[141,145]
**Flavones**				
Apigenin	hTAS2R14, hTAS2R39 (GI tract, respiratory)	Pathway: TAS2R → PLCβ2/IP_3_ → Ca^2+^ ↑; Nrf2/HO-1 Effect: ↓ oxidative damage; ↑ Nrf2/HO-1	TAS2R in vitro; antiox in vivo/in vitro	[141,149]
Luteolin	hTAS2R14, hTAS2R39 (GI tract, immune)	Pathway: TAS2R → PLCβ2/IP_3_ → Ca^2+^ ↑; Nrf2/HO-1/NQO1 Effect: ↑ Nrf2 targets; ↓ ROS	TAS2R in vitro; antiox in vivo/in vitro	[141,150,151]
Scutellarin	hTAS2R14, hTAS2R39 (GI tract)	Pathway: TAS2R → PLCβ2/IP_3_ → Ca^2+^ ↑; Nrf2/HO-1 Effect: ↓ oxidative stress; cytoprotection	TAS2R in vitro; antiox in vitro/in vivo	[141,152]
**Flavanones/Flavanonols**				
Eriodictyol	hTAS2R14, hTAS2R39 (GI tract)	Pathway: TAS2R → PLCβ2/IP_3_ → Ca^2+^ ↑; Nrf2/HO-1 Effect: ↓ oxidative stress; ↑ HO-1	TAS2R in vitro; antiox in vivo	[141,153]
Hesperetin	hTAS2R14, hTAS2R39 (GI tract, vascular)	Pathway: TAS2R → PLCβ2/IP_3_ → Ca^2+^ ↑; Nrf2-linked Effect: ↑ antioxidant genes; ↓ oxidative stress	TAS2R in vitro; antiox in vitro	[141,154]
Naringenin	hTAS2R14, hTAS2R39 (GI tract, immune)	Pathway: TAS2R → PLCβ2/IP_3_ → Ca^2+^ ↑; Nrf2/HO-1 Effect: ↓ ROS; improved redox/inflammation coupling	TAS2R in vitro; antiox in vivo/in vitro	[141,155]
Taxifolin	hTAS2R14, hTAS2R39 (GI tract, vascular)	Pathway: TAS2R → PLCβ2/IP_3_ → Ca^2+^ ↑; Nrf2/HO-1 Effect: ↓ oxidative damage; ↑ HO-1	TAS2R in vitro; antiox in vivo	[141,156,157]
**Flavonols**				
Fisetin	hTAS2R14, hTAS2R39 (GI tract, kidney)	Pathway: TAS2R → PLCβ2/IP_3_ → Ca^2+^ ↑; Nrf2/HO-1 Effect: ↓ oxidative stress	TAS2R in vitro; antiox in vivo	[141,158,159]
Isorhamnetin	hTAS2R14, hTAS2R39 (GI tract)	Pathway: TAS2R → PLCβ2/IP_3_ → Ca^2+^ ↑; Nrf2/Keap1-linked Effect: ↓ oxidative stress	TAS2R in vitro; antiox in vivo/in vitro	[141,160,161]
Kaempferol	hTAS2R14, hTAS2R39 (GI tract)	Pathway: TAS2R → PLCβ2/IP_3_ → Ca^2+^ ↑; Nrf2/HO-1/NQO1 Effect: ↓ oxidative stress; ↑ Nrf2 targets	TAS2R in vitro; antiox in vivo/in vitro	[141,162,163]
Myricetin	hTAS2R14, hTAS2R39 (GI tract, liver)	Pathway: TAS2R → PLCβ2/IP_3_ → Ca^2+^ ↑; Nrf2-linked Effect: ↓ MDA; ↑ SOD/GSH	TAS2R in vitro; antiox in vivo/in vitro	[141,164,165]
Quercetin	hTAS2R14, hTAS2R39 (GI tract, respiratory)	Pathway: TAS2R → PLCβ2/IP_3_ → Ca^2+^ ↑; Nrf2/Keap1/HO-1 Effect: ↓ oxidative injury; ↑ HO-1	TAS2R in vitro; antiox in vivo	[141,166,167]
**Isoflavones/Isoflavans**				
Biochanin A	hTAS2R14, hTAS2R39 (GI tract)	Pathway: TAS2R → PLCβ2/IP_3_ → Ca^2+^ ↑; Nrf2/HO-1 Effect: ↓ brain oxidative stress; ↑ Nrf2 nuclear translocation/HO-1	TAS2R in vitro; antiox in vivo	[168,169]
Daidzein	hTAS2R14, hTAS2R39 (GI tract)	Pathway: TAS2R → PLCβ2/IP_3_ → Ca^2+^ ↑; Nrf2-linked Effect: ↓ oxidative injury	TAS2R in vitro; antiox in vivo	[168,170,171]
Genistein	hTAS2R14, hTAS2R39 (GI tract)	Pathway: TAS2R → PLCβ2/IP_3_ → Ca^2+^ ↑; Nrf2/HO-1 Effect: ↓ oxidative stress; ↑ HO-1	TAS2R in vitro; antiox in vivo	[168,172]
(±)-Equol	hTAS2R14, hTAS2R39 (GI tract)	Pathway: TAS2R → PLCβ2/IP_3_ → Ca^2+^ ↑; NRF2/KEAP1 Effect: ↓ ROS; ↑ antioxidant enzymes	TAS2R in vitro; antiox mechanistic	[168,173]
**Anthocyanins**				
Malvidin-3-O-glucoside (M3G)	hTAS2R7 (GI tract)	Pathway: TAS2R → PLCβ2/IP_3_ → Ca^2+^ ↑; antioxidant defence Effect: ↓ oxidative stress; cytoprotection	TAS2R in vitro; antiox in vivo/in vitro	[139,174]
**Condensed tannins/Hydrolysable tannins**				
Procyanidin B2	hTAS2R5 (GI tract)	Pathway: TAS2R → PLCβ2/IP_3_ → Ca^2+^ ↑; Nrf2-linked Effect: ↓ ROS; ↑ antioxidant defence	TAS2R in vitro; antiox in vivo/in vitro	[139,175]
Procyanidin C2	hTAS2R5 (GI tract)	Pathway: TAS2R → PLCβ2/IP_3_ → Ca^2+^ ↑; antioxidant defence Effect: ↓ oxidative stress; ↓ lipid accumulation (model)	TAS2R in vitro; antiox in vivo/in vitro	[139,176]
Pentagalloylglucose (PGG)	hTAS2R5, hTAS2R39 (GI tract, immune)	Pathway: TAS2R → PLCβ2/IP_3_ → Ca^2+^ ↑; strong scavenging Effect: cytoprotection; ↓ ROS	TAS2R in vitro; antiox in vivo/in vitro	[139,177,178]

* Tissue column definition: tissues listed are examples of extraoral expression sites of the TAS2R subtype(s), not the antioxidant assay model. † EGCG: broader panels my report additional hTAS2Rs for EGCG; here we list the most consistently reported subtypes in catechin-focused TAS2Rs studies. Abbreviations: ARE, antioxidant response element; Ca^2+^, intracellular calcium; GI, gastrointestinal; GSH, reduced glutathione; HO-1, heme oxygenase-1; IP_3_, inositol-1,4,5-triphosphate; KEAP1, Kelch-like ECH-associated protein 1; MDA, malondialdehyde; NQO1, NAD(P)H quinone dehydrogenase 1; Nrf2/NRF2, nuclear factor erythroid 2-related factor 2; PLCβ2, phospholipase C-β2; ROS, reactive oxygen species; SOD, superoxide dismutase; TAS2R, type-2 bitter taste receptor. Legend: Canonical TAS2R signalling cascade: ligand binding → TAS2R activation → G protein coupling (gustducin/engineered Gα16gust44 in heterologous systems) → PLCβ2 activation → IP_3_ production → intracellular Ca^2+^ release (↑ Ca^2+^) → TRPM5 activation. Symbols: ↑, increased; ↓, decreased. *Abbreviations:* DAG, diacylglycerol; GLP-1, glucagon-like peptide-1; CCK, cholecystokinin; PLCβ2, phospholipase C-β2; TRPM5, transient receptor potential cation channel subfamily M member 5.

**Table 5:** Multi-level determinants of functional food acceptance: from biological markers to cognitive traits.

Influence	Determinant	Mechanism & Impact on Acceptance	Key References
Genetics	Taste Receptor *TAS2R38*	Polymorphisms (PAV vs. AVI) dictate bitter taste intensity. “Supertasters” (PAV) perceive functional ingredients as significantly more aversive.	[189,190]
	*CA6* Gene (Gustin)	Regulates fungiform papillae density. Variants are linked to increased tactile sensitivity, amplifying the perception of astringency/roughness.	[190]
Demographics	Biological Sex	Women generally exhibit higher fungiform papillae density and bitter responsiveness, increasing susceptibility to sensory rejection of antioxidants.	[196]
Oral Physiology	Salivary Pellicle Dynamics	Interaction with tannins causes selective depletion of lubricating peptides (e.g., statherin, P-B), disrupting the mucosal pellicle and exposing mechanoreceptors to friction.	[193]
	Host–Microbiome Ecology	Host genetics (AMY1, FUT2, ABO) shape the oral ecosystem. Specific microbiotypes hydrolyse aroma precursors, creating personalised “in-mouth” flavour profiles.	[56,194]
	Salivary Flow & Ageing	Reduced salivary flow in the elderly compromises the mucosal pellicle, increasing friction and the persistence of “dry” sensations despite higher sensory thresholds.	[195]
Nutritional Status	BMI & Inflammation	Obesity-related inflammation reduces taste bud abundance (“blunted” sensitivity), requiring higher stimulus intensity to elicit hedonic reward.	[197,198]
Neurobiology	Prefrontal Cortex (DLPFC)	Hemodynamic responses in the right DLPFC track the intensity of astringency, while the left DLPFC encodes hedonic evaluation, linking oral biophysics directly to neural “value” calculation.	[201]
Sensory Dynamics	Temporal Carry-over	Accumulation of astringency over repeated sips leads to palate fatigue and negative emotional responses before consumption is complete.	[186]
Psychology	Neophobia & Punishment (SP)	High “Food Neophobia” and “Sensitivity to Punishment” scores drive the rejection of novel/bitter profiles due to fear of adverse consequences.	[187]
	Sweet Liking Phenotypes	“High Sweet Likers” reject bitterness without sugar masking; “Inverted-U” phenotypes show higher baseline acceptance of unsweetened functional foods.	[188]
Cognition & Learning	Sustainability Halo	Cognitive cues (e.g., “eco-friendly”) raise the tolerance threshold for off-flavours, allowing consumers to accept a conscious “sensory trade-off”.	[185,202]
	Flavour-Nutrient Learning	Metabolic feedback (e.g., caffeine stimulation) recodes “bitter” taste from a warning signal to a predictor of reward, reinforcing intake even in genetically sensitive individuals.	[203,204]

## Data Availability

No new data were created or analyzed in this study. Data sharing is not applicable to this article.

## Supplementary Materials

The following supporting information can be downloaded at https://www.mdpi.com/article/10.3390/antiox15030397/s1, Figure S1: Bibliometric network map of keyword co-occurrence based on the literature cited in this review.

## Abbreviations

The following abbreviations are used in this manuscript:

aPRP	Acidic Proline-Rich Protein
BA9	Brodmann Area 9
BAS	Behavioural Activation System
BIS	Behavioural Inhibition System
cAMP	Cyclic Adenosine Monophosphate
CCK	Cholecystokinin
DLPFC	Dorsolateral Prefrontal Cortex
DNA	Deoxyribonucleic acid
EC	(-)-Epicatechin
ECG	(-)-Epicatechin Gallate
EGC	Epigallocatechin
EGCG	Epigallocatechin Gallate
fNIRS	Functional Near-Infrared Spectroscopy
FN	Food Neophobia
GI	Gastrointestinal
GLP-1	Glucagon-Like Peptide-1
gPRP	Glycosylated Proline-Rich Protein
GSH	Reduced Glutathione
GSPE	Grape Seed Proanthocyanidin Extract
HA	Hyaluronic Acid
HO-1	Heme Oxygenase-1
IL-6	Interleukin-6
IP_3_	Inositol Trisphosphate
Keap1	Kelch-like ECH-associated protein 1
LCT	Long-Chain Triglyceride
M3G	Malvidin-3-*O*-glucoside
MCT	Medium-Chain Triglyceride
MDA	Malondialdehyde
mDP	Mean Degree of Polymerisation
MRP2	Multidrug Resistance-Associated Protein 2
NEPPs	Non-Extractable Polyphenols
NF-κB	Nuclear Factor Kappa B
NLRP3	NLR Family Pyrin Domain Containing 3
NO	Nitric Oxide
NQO1	NAD(P)H Quinone Dehydrogenase 1
Nrf2	Nuclear Factor Erythroid 2-Related Factor 2
OVA	Ovalbumin
P-gp	P-glycoprotein
PKA	Protein Kinase A
PLCβ2	Phospholipase C Beta 2
PROP	6-n-propylthiouracil
PRP	Proline-Rich Protein
PYY	Peptide YY
ROS	Reactive Oxygen Species
SAR	Structure-Activity Relationship
SNP	Single Nucleotide Polymorphism
SOD	Superoxide Dismutase
SP	Sensitivity to Punishment
SR	Sensitivity to Reward
TAS2R	Type 2 Taste Receptor (Human Bitter Taste Receptor)
Tas2r	Type 2 Taste Receptor (Rodents Bitter Taste Receptor)
TDS	Temporal Dominance of Sensations
TNF-α	Tumour Necrosis Factor Alpha
TPO	Thyroide Peroxidase
TRPM5	Transient Receptor Potential Cation Channel Subfamily M Member 5
