# Supplementary material for: Beyond Sensory Properties: Molecular Interactions of Antioxidant Flavour-Active Polyphenols Across the Food-Oral-Gut Axis
Source: Antioxidants (Basel). 2026 Mar 21;15(3):397. doi: 10.3390/antiox15030397 (PMC13023888; doi:10.3390/antiox15030397)
Supplement: Supplementary file 1 [file antioxidants-15-00397-s001.zip › antioxidants-4198388-supplementary.pdf]

Supplementary Materials

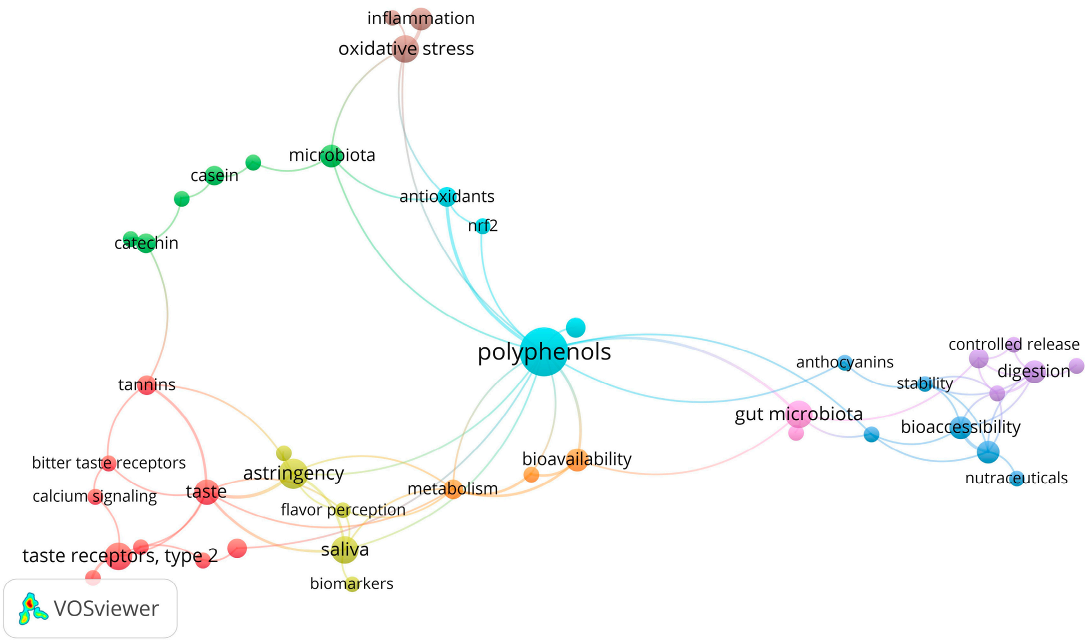

Supplementary Figure S1. Bibliometric network map of keyword co-occurrence based on the literature cited in this review. The analysis was performed using VOSviewer software to illustrate the interdisciplinary connections across the food-oral-gut axis. Node size represents the frequency of keyword occurrence, while line thickness indicates the strength of the association between terms.
